# The benefits, challenges, and best practice for patient and public involvement in evidence synthesis: A systematic review and thematic synthesis

**DOI:** 10.1111/hex.13787

**Published:** 2023-06-01

**Authors:** Eldad Agyei‐Manu, Nadege Atkins, Bohee Lee, Jasmin Rostron, Marshall Dozier, Maureen Smith, Ruth McQuillan

**Affiliations:** ^1^ Centre for Population Health Sciences, Usher Institute University of Edinburgh Edinburgh Scotland UK; ^2^ Asthma UK Centre for Applied Research University of Edinburgh Edinburgh Scotland UK; ^3^ National Institute for Economic and Social Research London UK; ^4^ Cochrane Consumer Network Executive Ottawa Ontario Canada

**Keywords:** benefits, best practice, challenges, evidence synthesis, patient and public involvement, systematic review, thematic synthesis

## Abstract

**Introduction:**

Despite the growing evidence on patient and public involvement (PPI) in health research, little emphasis has been placed on understanding its quality and appropriateness to evidence synthesis (ES) and systematic reviews (SR). This study aimed to synthesise qualitative evidence on the benefits, challenges, and best practices for PPI in ES/SR projects from the perspectives of patients/public and researchers.

**Methods:**

We searched Ovid MEDLINE, Ovid EMBASE, Cochrane Library and CINAHL Plus. We also searched relevant grey literature and conducted hand‐searching to identify qualitative studies which report the benefits and challenges of PPI in individual ES/SR projects. Studies were independently screened by two reviewers for inclusion and appraised using the Joanna Briggs Institute's Qualitative Tool. Included studies were synthesised narratively using thematic synthesis.

**Results:**

The literature search retrieved 9923 articles, of which eight studies were included in this review. Five themes on *benefits* emerged: two from patients'/public's perspective—gaining knowledge, and empowerment; and three from researchers' perspective—enhancing relevance, improving quality, and enhancing dissemination of findings. Six themes on *challenges* were identified: three from patients'/public's perspective—poor communication, time and low self‐esteem; and three from researchers' perspective—balancing inputs and managing relations, time, and resources and training. Concerning recommendations for best practice, four themes emerged: provision of sufficient time and resources, developing a clear recruitment plan, provision of sufficient training and support, and the need to foster positive working relationships.

**Conclusion:**

Highlighting the benefits and challenges of PPI in ES/SR projects from different stakeholder perspectives is essential to understand the process and contextual factors and facilitate meaningful PPI in ES/SR projects. Future research should focus on the utilisation of existing frameworks (e.g., Authors and Consumers Together Impacting on eVidencE [ACTIVE] framework) by researchers to help describe and/or report the best approaches and methods for involving patients/public in ES/SRs projects.

**Patient and Public Contribution:**

This review received great contributions from a recognised PPI partner, the Chair of the Cochrane Consumer Network Executive, to inform the final stage of the review (i.e., interpretation, publication and dissemination of findings). The PPI partner has been included as an author of this review.

## 1 INTRODUCTION

Involving patients/public in research is increasingly regarded as essential to achieving high‐quality, relevant, patient‐centred research outputs which contribute to improving health and facilitating sustainable health service delivery.^1^ Patient and public involvement (PPI) describes the active process of effective partnership working with patients and members of the public to contribute to how health research is planned, managed, designed, conducted and disseminated.^2^ It is well‐established in high‐income countries such as Canada, the United States of America and the United Kingdom,^3^ where compelling ethical and political discourses emphasise the rights of patients/public to have a ‘voice’ in public services,^4^, ^5^ as well as the pragmatic considerations and policy response in identifying involvement as a condition for research funding,^6^ and the potential for PPI to enhance the relevance of research outputs.^7^

The 7Ps framework of stakeholder engagement identifies and categorises various stakeholders involved in health research as follows: providers, policymakers, payers, purchasers, product makers, principal investigators (e.g., researchers) and patients/public.^8^ This framework serves as a guide for researchers on which specific stakeholder(s) to involve in research project(s), as well as the stakeholders' roles/responsibilities in these research project(s). The UK's National Institute for Health and Care Research (NIHR) defines patients/public as ‘patients, potential patients, carers and people who use health and social care services, people from organisations that represent people who use services’.^9^ Notably, patients/public are important stakeholders in research priority setting and funding.^10^ However, notwithstanding the positive strides made towards stakeholder involvement in health research, there is still uncertainty about how researchers might best involve patients/public in different types of research and how the impact of their involvement on the conduct and quality of research should be evaluated.^11^

Evidence synthesis (ES), also referred to as systematic review (SR), a rigorous and widely used research methodology in public health and medical research, seeks to produce the least biased answer possible to a specific clinical or public health question, by identifying, critically evaluating and synthesising all the relevant evidence on the topic. In recent times, there have been calls for greater inclusion of patients/public and other relevant stakeholders in ES to enhance transparency and improve the relevance of research addressing public and policy health priorities.^12^, ^13^ The potential integration of patients/public into evidence‐informed health research is vital to enhancing desired patient‐centred health outcomes, improving the public's confidence in the healthcare system, and making informed decisions about the care of individual patients.^14^ The NIHR's UK Standards for Public Involvement^15^ provides a framework for effective public involvement in research in general. It underpins how researchers could use this framework to review their plans for PPI, as well as how research funders could assess the implementation of PPI in research projects in general. The Authors and Consumers Together Impacting on eVidencE (ACTIVE) framework,^16^ however, focussed specifically on how to involve diverse stakeholders in SRs. The ACTIVE framework provides a structure that summarises key components of the engagement of knowledge users in SRs, and this is becoming increasingly influential in shaping Cochrane's approach to involvement. Our review builds on the existing ACTIVE framework by enhancing our understanding of best practices for researchers to involve *specific stakeholders* (i.e., patients/public) in an ES/SR project, considering the perspectives of both researchers and patients/public.

A study by Cottrell et al.^17^ provided evidence on the benefits and challenges of stakeholder engagement in SRs and reported key themes on benefits (such as ‘ensuring transparency and accountability’, ‘establishing credibility’, etc.) and challenges (such as ‘time’, ‘finding the right people’, etc.). However, the ‘benefits’ as defined in this study did not capture the personal benefits of involvement, the benefits of training stakeholders about the research process, and the benefits of fostering relationships. Furthermore, whilst this paper explored the benefits and challenges from stakeholders' perspective *in general*, to the best of our knowledge, there is no qualitative ES reporting on the benefits and challenges of PPI in ES/SRs from the perspectives of *specific groups of stakeholders* (i.e., researchers and patients/public). The current review, therefore, aims to address this gap in the literature. This paper aims to

1. Synthesise qualitative evidence on the benefits and challenges of PPI in individual ES/SR projects from the perspectives of patients/public and researchers.
2. Synthesise qualitative evidence on the best practice(s) for researchers to effectively involve patients/public in individual ES/SR projects.

## 2 METHODS

The reporting of this qualitative SR followed the Enhancing Transparency in Reporting the Synthesis of Qualitative Research (ENTREQ) framework.^18^

### 2.1 Operational definition of terms

Due to the conceptual differences and the lack of a consensus on the terms used to describe PPI in research,^19^, ^20^ the following operational definitions were used in this review:

1. ‘Patients/public’ and ‘PPI’ are defined according to the UK's NIHR's definitions for ‘the public’ and ‘public involvement’, respectively.^9^ Thus, patients/public refers to ‘patients, potential patients, carers and people who use health and social care services, and people from organisations that represent people who use services’. PPI denotes ‘research being carried out “with” or “by” members of the public, rather than “to”, “about” or “for” them’.
2. ‘Benefits’ is defined as the potential positive impact/influence on the quality, conduct and dissemination of ES/SR projects and/or the opportunities associated with involving individuals in the synthesis/review process. Thus, describing personal impact on individuals and impact on synthesis/review studies.
3. ‘Challenges’ is defined as the problems, difficulties or barriers related to PPI in ES/SR projects. These may include personal, organisational or behavioural difficulties.
4. ‘Best practice’ is defined as the effective ways of meaningfully involving patients/public in the synthesis/review process, as recommended by the authors of included papers.
5. The terms ‘evidence synthesis’ and ‘systematic review’ are used interchangeably to explain the process of using a systematic, explicit method to review evidence from primary research; although there are debates surrounding the use of these terms.^21^

### 2.2 Search strategy

To define the key elements of the research questions and inform the standardisation of the search strategy, the SPIDER (Sample, Phenomenon of Interest, Design, Evaluation, Research type) tool was chosen^22^ (see Table 1) to identify relevant qualitative and/or mixed‐method studies. However, due to poor indexing of qualitative studies in databases, the use of descriptive titles/abstracts, poor description of qualitative methods^23^, ^24^ and limited time, the decision was made that the search strategy would be constructed by identifying and combining keywords/concepts supporting this review's aims/objectives^25^, ^26^: (i) PPI (e.g., patient, citizen, public; and engagement, involvement, participation); and (ii) ES (e.g., SR [as both subject and publication type], evidence‐based reviews); and (iii) healthcare research (e.g., health service, health research, clinical research).

**Table 1:** Defining review questions using the SPIDER tool.

Sample	Researchers and patients/public
Phenomenon of interest	PPI in ES/SRs.
Design	Studies that utilised qualitative data collection methods and analysis.
Evaluation	Perspectives on benefits, challenges, and best practice.
Research types	Qualitative or mixed methods studies describing/providing qualitative data.

Abbreviations: ES, evidence synthesis; SR, systematic reviews.

The predetermined, systematic search was conducted in four bibliographic databases: Ovid EMBASE, Ovid MEDLINE, Cochrane Library and the Cumulative Index to Nursing and Allied Health Literature (CINAHL Plus). To supplement the bibliographic database searches, grey literature was searched from relevant databases and websites: ‘INVOLVE Libraries: public involvement in research’, Grey Literature Report (GreyLit), Patient‐Centered Outcomes Research Institute (PCORI), Centre for Reviews and Dissemination (CRD), and the Evidence for Policy and Practice Information and Co‐ordinating Centre (EPPI‐Centre). Furthermore, the reference lists of key studies were screened for additional articles.^27^ There were no restrictions on publication status, language or publication date before conducting the searches to avoid bias.^28^ See Supporting Information: File S1 for search strategy details.

### 2.3 Study selection

Two independent reviewers (E. A. M. and N. A.) systematically selected studies for inclusion via the Covidence software,^29^ based on the eligibility criteria outlined below. Titles and abstracts of all studies were first reviewed, followed by a full‐text review of eligible studies. Any disagreements were resolved in consultation with a third reviewer (R. M.).

For studies to be included in this review, they had to meet the following criteria:

1. Studies or documents (e.g., reports from health institutions) which describe, reflect on, or evaluate the perspectives of patients/public and/or researchers on the benefits and challenges of PPI in individual ES/SR projects.
2. Studies or documents written by researchers or together with patients/public providing evidence or detailing their experiences in individual ES/SR projects.
3. Studies or documents about ES/SRs where patients/public were involved in at least one stage of the ES/SR process.
4. Studies or documents written in the area of health research.
5. Studies or documents written in English language and with full text.

Studies were excluded from this review if they described patients'/public's and/or researchers' involvement in other types of research (e.g., clinical trials), and/or were limited to research in social science. Commentaries, editorials, conference abstracts and letters were also excluded.

### 2.4 Quality assessment

The Joanna Briggs Institute's (JBI) qualitative checklist was used to assess the methodological quality of included studies.^30^ Quality of studies were graded as follows: ‘High’—80% or greater; ‘Moderate’—greater than 50% but less than 80%; and ‘Low’—less than 50%. Quality assessment was conducted independently by two reviewers (E. A. M. and N. A.). Any disagreements were resolved in consultation with a third reviewer (R. M.).

Additionally, the overall confidence in the evidence obtained in this qualitative SR was evaluated using the Grading of Recommendations, Assessment, Development and Evaluation—Confidence in Evidence from Reviews of Qualitative Research (GRADE‐CERQual) approach.^31^

### 2.5 Data extraction

A data extraction template was developed, piloted and appropriately refined using two included studies. Extracted data included author name (and year); study title; location/country; study aims/objectives; study design/type; health topic/focus; description of patients/public involved; stage, level, role, and/or method of PPI; description of the benefits and challenges of PPI from the perspectives of patients/public and researchers; authors' recommendations for best practice or the challenges identified (see Table 3 and Supporting Information: Files S4 and S5).

**Table 2:** Guidance for reporting involvement of PPI partner (GRIPP2 checklist).

Aim	Our aim was to synthesise qualitative evidence on the benefits, challenges and best practices for PPI in ES/SR projects from the perspectives of patients/public and researchers and work collaboratively with a PPI partner to analyse the findings, contribute to the writing of the report, and the publication and dissemination of findings.
Methods	The PPI partner was invited via a closed recruitment strategy. Authors (E. A. M. and R. M.) met virtually with M. S. to present the review and the journal reviewers' feedback and discuss whether there was agreed‐upon interest to contribute to this review and how this involvement could meet everyone's needs. Several virtual consultative meetings and email communications between authors (E. A. M. and R. M.) and M. S. ensued. M. S. contributed to the final draft of the review at all stages of the writing of the report and the responses to the reviewers. Authors (E. A. M. and R. M.) were committed to supporting M. S.'s contributions. The PPI partner was included as a coauthor of this review due to her involvement and contributions which meet the ICMJE authorship criteria.
Study results	The PPI partner provided valuable input and understanding in interpreting the findings of the review and in providing context based on her experiences as a PPI coproducer of ES/SRs. The PPI partner led the development of the content for the GRIPP2 checklist.
Discussion and conclusions	PPI partner highlighted important patients'/public's perspectives on involvement processes and provided valuable input in drawing appropriate conclusions from the evidence generated in this review.
Reflections/critical perspective	The study authors, who did not have prior experience with PPI in ES/SRs, learned much from M. S. regarding the methods and value of PPI. As a result, they see the value of involving patients/public throughout the lifecycle of synthesis/review projects and are confident that this involvement can become an essential part of their research methods on ES/SRs. M. S. benefitted from collaborating with a team who are willing to try new approaches, which was of value to her goal of advancing coproduction of ES/SRs.

Abbreviations: ES, evidence synthesis; GRIPP2, Guidance for Reporting Involvement of Patients and the Public; PPI, patient and public involvement; SR, systematic reviews.

To preserve the unique perspectives being presented by authors, original and quoted texts from the selected papers describing the benefits, challenges, and best practice(s) for PPI were directly extracted. Due to the possible distortion of data as ‘findings’ (e.g., when participants' data assumingly ‘speak for themselves’ without researchers’ interpretation) and the different reporting styles in qualitative research,^32^ ‘findings’ from selected studies relating to the *benefits*, *challenges* or *best practice* were considered to be all original and quoted text were labelled under the ‘Abstract’ and/or ‘Results’ sections in the study reports. In instances where similar texts were provided by authors in both the ‘Abstract’ and ‘Results’ sections, the text from the ‘Results’ section was extracted—to enhance the contextual meaning of the data being extracted. Additionally, texts were included from the ‘Discussion’ section of study reports if they contained extra valuable information relating to *benefits*, *challenges* or *best practices*. Extraction was completed by E. A. M. and independently checked by N. A. Any disagreements were resolved in consultation with a third reviewer (R. M.).

### 2.6 Data analysis and synthesis

We utilised the methods of thematic synthesis by Thomas and Harden^33^ to enhance transparency in the synthesis of extracted data. Data from included studies were gathered into a Word document for coding. One author (E. A. M.) manually conducted an inductive line‐by‐line coding of participant (i.e., patients/public and researchers) accounts from two included studies. The set of codes was further developed as the remaining included studies were added and verified through repeated discussions with another author (J. R.). Based on the content of the codes, one author (E. A. M.) identified sets of related codes and organised them into 15 broad descriptive themes (see Supporting Information: File S6). One author (E. A. M.) drafted a summary of the 15 descriptive themes across the included studies. Two other authors (J. R. and R. M.) independently commented on this draft—after repeated reference back to the included papers—and a final version was agreed upon. The descriptive themes related to the perspectives of patients/public and researchers on their involvement in ES/SR projects. The final summary was then used by the authors (E. A. M., J. R. and R. M.) to identify analytical themes emerging from the descriptive themes across the included studies, using the research objectives as a framework for interpretation.

### 2.7 Patient and public involvement

A recognised PPI partner (M. S.), Chair of the Cochrane Consumer Network Executive, was invited to contribute to the final stage of the review—interpretation of review findings, writing of the review, and the publication and dissemination of findings. Consultative meetings between authors (E. A. M. and R. M.) and the PPI partner were held online. The reporting of PPI in this review was guided by the revised Guidance for Reporting Involvement of Patients and the Public (GRIPP2) checklist^34^—the first international guidance for reporting of PPI in health and social care research—to enhance the transparency and quality of the PPI evidence base. Table 2 summarises PPI in our review.

## 3 RESULTS

### 3.1 Search results

**Figure 1:**
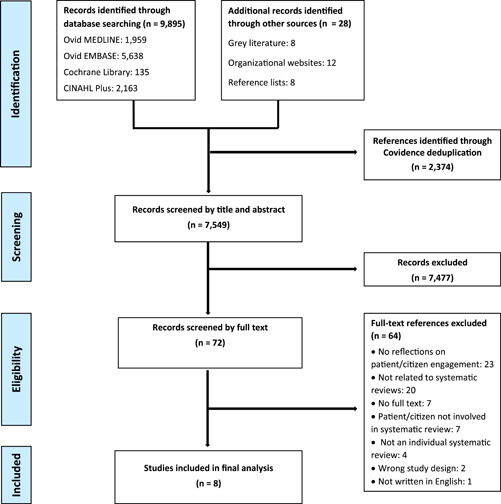
PRISMA flowchart showing the selection of relevant literature.

Overall, 9923 titles and abstracts were screened, and 72 studies were independently assessed for eligibility during the full‐text screening stage. A total of eight studies^35^, ^36^, ^37^, ^38^, ^39^, ^40^, ^41^, ^42^ were included in the final analysis (see Figure 1).

### 3.2 Characteristics of included studies

Table 3 summarises the characteristics of the eight studies included in this review. Seven of these studies were entirely or mainly conducted within the United Kingdom. All included studies were qualitative, peer‐reviewed articles that focussed on patient‐related or public health‐related topics and were published between 2012 and 2021.

**Table 3:** Characteristics of included studies.

No.	Author (year)	Study title	Country	Study aim(s)	Study design	Health topic and focus	Description of patients/public involved (including demographics)	Method, stage, role, and level of PPI
1.	Bayliss et al. (2016)^35^	‘Patient involvement in a qualitative meta‐synthesis: lessons learnt’.	UK, Sweden, Estonia and Romania	‘…to evaluate the patient experience of involvement in a qualitative meta‐synthesis’.	Open‐ended questionnaire via email	Chronic inflammatory diseases. Reported on the benefits and challenges from patients'/public's perspective, and on recommendations for best practice.	8 PRPs who were involved in the EuroTEAM study.	Consultation: Focus sessions with PRPs during analysis and interpretation stages of the review process. Collaboration: Invitation by email to contribute to writing grant application and development of search strategy, and initial validation of coding during analysis stage (metasynthesis).
2.	Coon et al. (2016)^36^	‘End‐user involvement in a systematic review of quantitative and qualitative research on nonpharmacological interventions for attention deficit hyperactivity disorder delivered in school settings: reflection on the impacts and challenges’.	UK	To: ‘highlight the methods of end‐user involvement used in our reviews’. ‘facilitate and stimulate discussion of the most appropriate and efficient methods of engagement’. ‘develop a list of suggestions to improve future involvement in systematic reviews’.	Unclear	‘Nonpharmacological interventions for attention deficit hyperactivity disorder’. Reported on the benefits and challenges from patients'/public's and researchers' perspectives, and on recommendations for best practice.	‘Families of children and young people with ADHD; teaching, special educational needs, and mental health services professionals; researchers’.	Consultation: Four workshops held with parents and carers of children with ADHD, researchers, behavioural support professionals, and teaching professionals. Collaboration: A behavioural support professional, a parent of children with ADHD, and members of the Expert Advisory Group were involved in designing the project, protocol development, organising events, providing feedback on the final report via email contact or face‐to‐face meetings.
3.	Hyde et al. (2017)^37^	‘Process and impact of patient involvement in a systematic review of shared decision making in primary care consultations’.	UK	‘…to describe the process and impact of involving patients in a systematic review and narrative synthesis of shared decision making around prescribing analgesia for musculoskeletal pain in primary care consultations’.	Workshops and [focus] group discussion	‘Prescribing analgesia for musculoskeletal pain’. Reported on the benefits and challenges from researchers' perspectives, and on recommendations for best practice.	5 RUG members of both sexes and with varying ages; a RUG coordinator and a user support worker.	Consultation: Three workshops held with RUG members to explore their understanding of systematic reviews, and plan and discuss how to share the results. Collaboration: RUG members were involved during the designing of protocol, results interpretation, and designing dissemination strategies via workshops held at three time‐points.
4.	Jamal et al. (2015)^38^	‘Consulting with young people to inform systematic reviews: an example from a review on the effects of schools on health’.	UK	‘…to describe the process and impact of consulting with a young people's advisory group to inform decision making in a systematic review on the effects of schools and school environment interventions on children and young people's health’.	[focus] group discussions	‘Effects of schools and school environment interventions on young people's health’. Reported on the benefits and challenges from researchers' perspectives.	‘25 young people (aged 14–19 years) from Bristol and South Wales; predominantly White and middle class, with small number of ethnic minorities’.	Consultation: Two face‐to‐face meetings with young people's advisory group (ALPHA) during project inception and review mapping stages; augmented with online discussion.
5.	Oliver et al. (2015)^39^	‘Broadening public participation in systematic reviews: a case example involving young people in two configurative reviews’.	UK	To: ‘describe the approaches used to elicit young people's views in relation to topics and draft findings in two configurative systematic reviews’. ‘describe the perspectives that were elicited and the extent to which these influenced the reviews’. ‘reflect on how well these approaches worked and what this might mean for others planning similar involvement activities’.	Workshops and group discussions	Childhood obesity. Reported on the benefits and challenges from researchers' perspectives.	Young people (PEAR group members) aged 12–17 years.	Consultation: Two workshops with young people's advisory group to check the reliability of synthesis and develop implications of the review (in the views review) and identify factors associated with educational attainment and obesity during discussion of preliminary findings (in the correlational review).
6.	Troya et al. (2019)^40^	‘Patient and public involvement and engagement in a doctoral research project exploring self‐harm in older adults’.	UK	‘…to critically reflect on the process, potential impact and identify challenges/opportunities in involving robust patient public involvement and engagement in a doctoral research, including a systematic review and qualitative study’.	Workshops and group discussions	Self‐harm in older adults. Reported on the benefits and challenges from researchers' perspectives, and on recommendations for best practice.	PPIE group comprised of ‘an older female adult with self‐harm history, a male carer, and a female support worker with previous experience of self‐harm; all aged ≥60 years’.	Collaboration and ongoing consultation: Four workshops held with PPIE members to refine the review's scope, refining search strategy and eligibility criteria, clarify analysis and interpretation, and to seek advice on dissemination of findings.
7.	Vale et al. (2012)^41^	‘Evaluation of patient involvement in a systematic review and meta‐analysis of individual patient data in cervical cancer treatment’.	UK	‘…to evaluate [patient research partners] involvement with the aim of informing the practice of patient involvement in future systematic reviews’.	Individual, open‐ended questionnaire	Treatments for cervical cancer. Reported on the benefits and challenges from patients'/public's and researchers' perspectives, and on recommendations for best practice.	Six women (PRPs) recruited, but three were involved in the evaluation.	Consultation: Establishment of a small reference group to offer guidance on recruitment, support, and project's activities. Collaboration: PRPs were involved during the stages of locating study investigators, interpretation of findings, and writing of an editorial on review's findings.
8.	Walker et al. (2021)^42^	‘No evidence synthesis about me without me: involving young people in the conduct and dissemination of a complex evidence synthesis’.	UK	‘…to describe, from the researcher team, the process of engaging and working alongside children and young people with long‐term physical health conditions and mental health difficulties and their parents whilst conducting and disseminating a complex evidence synthesis’.	Workshops and [focus] group discussions	Long‐term conditions in children and young people. Reported on the benefits from patients'/public's perspective, benefits and challenges from researchers' perspective, and on recommendations for best practice.	8 CYP (10–17 years), three were females; CYP lived with rheumatic or neurological LTCs Five parents attending meetings.	Collaboration: CYP were involved in four face‐to‐face meetings while conducting evidence synthesis, and three dissemination events.

Abbreviations: ADHD, attention deficit hyperactivity disorder; ALPHA, Advice Leading to Public Health Advancement; CYP, children and young people; CYPAG, Children and Young People's Advisory Group; EuroTEAM, Towards Early biomarkers in Arthritis Management; LTC, long‐term condition; NCB, National Children's Bureau; PARE, People with Arthritis/Rheumatism in Europe; PEAR, ‘Public health, Education, Awareness, Research’; PPI, patient and public involvement; PPIE, patient and public involvement and engagement; PRP, patient research partner; RA, rheumatoid arthritis; RUG, research user group.

### 3.3 Quality assessment

#### 3.3.1 Using the JBI qualitative checklist

**Figure 2:**
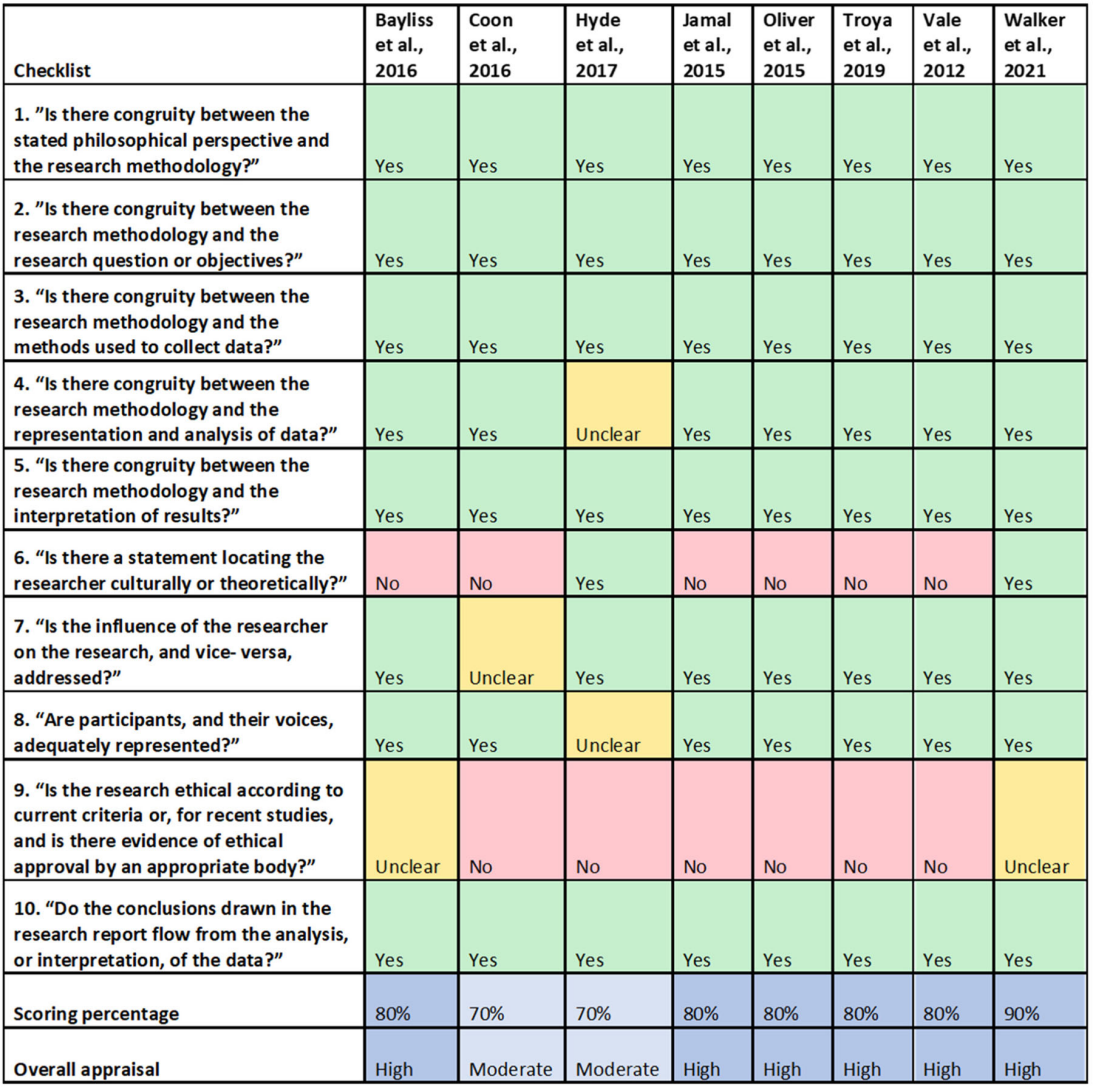
Quality assessment of included studies using the Joanna Briggs Institute's Qualitative tool.

Based on the calculation of the quality score for each included study, six studies were considered to be of high quality^35^, ^38^, ^39^, ^40^, ^41^, ^42^ while the remaining two were of moderate quality^36^, ^37^ (see Figure 2).

#### 3.3.2 Using the GRADE‐CERQual approach

Based on the assessment of methodological limitations, coherence, relevance and adequacy, the overall confidence in the findings of this review was rated as moderate. Thus, it is possible that the findings of this review reasonably represent the true phenomenon. The summary of qualitative findings using the GRADE‐CERQual approach is presented in Supporting Information: File S3.

### 3.4 Benefits of PPI in ES/SRs

#### 3.4.1 The perspective of patients/public

##### Gaining knowledge

Patients/public who received training and support as part of the research process felt that they had either improved their level of knowledge about ES/SRs in general or about the specific topic/study that they were involved in.^35^, ^41^ They acquired skills such as coding, searching, listening, writing summaries, and interpreting results.^35^, ^41^ Additionally, patients/public felt that their partnership with other members of the review team enabled them to share useful ideas and learning experiences about the synthesis/review project.^35^, ^41^, ^42^

*Taking into consideration that it was the first activity of that kind in my experience as a Patient Research Partner, I was very happy and thankful to gain experience in coding. I improved my skills in summarising, finding keywords and interpreting. I gained confidence in participating in future coding activities. It was also very interesting to see how other people think about rheumatoid arthritis and prevention—very educative*. (Patient research partner 1)^35^

Patients'/public's open attitude towards ES/SRs aided them to discover pieces of information relevant to the synthesis/review project and thus enabled them to demystify ES/SRs and contribute effectively to the outcomes of the current projects^35^, ^41^ and future projects.^35^, ^41^, ^42^

##### Empowerment

Patients/public felt their confidence in shaping the review projects had improved due to their participation in the synthesis/review activities and after hearing the perspectives of other review team members.^35^, ^42^ They gained a real sense of achievement by either making positive changes to the individual ES/SR projects or by making valuable contributions to the scientific community.^35^, ^36^, ^41^, ^42^

*…being able to provide some information about late effects and pulling together our thoughts on these issues and getting both in print has been a great achievement… the outcome passed my expectations, as I witnessed how the team doing the evaluation realised what information was missing and how future trials could give more detailed and long‐term information*.^41^

Furthermore, patients/public felt that their involvement also created empowering relationships between researchers and themselves, which ensured the provision of mutual support^35^, ^41^, ^42^ and offered the opportunity to shape future attitudes of patients/public towards ES/SR projects.^35^, ^36^

#### 3.4.2 The perspective of researchers

##### Improving quality

Researchers felt that they were able to identify the specific scientific areas or stages in the ES/SR process where patients'/public's contributions enhanced the quality of the final report, including refining the scope of the review,^36^, ^37^, ^38^, ^40^ defining research aims and questions,^38^, ^40^ informing search strategies,^36^, ^37^, ^38^, ^40^, ^42^ developing review protocol,^36^, ^37^, ^40^ informing data extraction and analysis,^36^, ^37^, ^39^, ^40^, ^41^, ^42^ interpreting review findings,^36^, ^37^, ^38^, ^39^, ^40^, ^42^ and writing of the final report and developing implications for research and practice.^36^, ^39^ Researchers saw the perspectives of patients/public during the technical aspects of the synthesis/review process (e.g., data analysis, interpreting themes/findings) to be very useful because patients/public had received prior training in undertaking research activities.^38^, ^41^ Most researchers highlighted that PPI in the synthesis/review process was valuable in identifying gaps in evidence/literature, which otherwise would have been missed by researchers,^36^, ^37^, ^38^, ^39^, ^40^, ^41^, ^42^ and helped develop recommendations for future ES/SR projects.^36^, ^37^, ^40^, ^41^, ^42^

*The [patient and public involvement and engagement] group also identified gaps in the literature from the systematic review which they considered as important for patients and public and which require further research*.^40^

##### Enhancing relevance

Researchers felt that they were able to gain an accurate and deeper understanding of health issues related to the ES/SR project,^36^, ^38^, ^40^ how patients/public think and feel,^38^, ^40^, ^42^ and obtained a better understanding of the health needs of the patients/public.^36^, ^37^, ^38^, ^40^ Researchers agreed that involving patients/public enabled them to identify an unknown but critical perspective, which otherwise would have been missed by researchers, especially during the interpretation of synthesis/review findings.^38^, ^39^, ^40^, ^41^

*The researchers felt the greatest impact of involving the Patient Research Partners was the insight they gained of the impact of cervical cancer, its treatments and side effects, on the women's day‐to‐day lives, which would not have been possible without the Patient Research Partner involvement*.^41^

This enabled researchers to help identify relevant outcomes in the synthesis/review projects^36^, ^38^, ^42^ by incorporating patients'/public's priorities in the review process to address the real‐world concerns that they had.^37^, ^38^, ^40^ Generally, researchers agreed that they had greater confidence in the results/outcomes obtained in the ES/SR.^36^, ^39^, ^40^ Additionally, researchers noted that involving patients/public was useful in informing future ES/SR projects.^37^, ^40^, ^41^, ^42^

##### Enhancing dissemination of findings

Researchers agreed that patients/public played a vital role in planning and developing appropriate strategies and activities to disseminate findings and maximise their impact.^37^, ^40^, ^42^

*Results were targeted at practitioners, as RUG [research user group] members felt [that] this was most important. RUG members and support team participated in the dissemination of the review findings*.^37^

Researchers felt that they were able to develop dissemination strategies (including publishing editorials, producing podcasts, conference presentations and writing lay summaries) in consultation with the patients/public involved, which were targeted at different stakeholders, including patients, practitioners, policymakers and interested organisations.^37^, ^40^, ^42^

### 3.5 Challenges of PPI in ES/SRs

#### 3.5.1 The perspective of patients/public

##### Poor communication

Patients/public reported that the use of ambiguous terms/language by researchers while explaining key aspects of the ES/SR project and its related literature and processes during training sessions was a barrier.^35^, ^41^ This made them feel that they lacked the practical skill and knowledge required to successfully undertake the synthesis/review project.^35^, ^36^, ^41^

*It was very difficult to comprehend fully research documents dealing with conditions other than RMDs [Rheumatic and Musculoskeletal Diseases] without an appropriate glossary etc*. (Patient research partner 4)^35^

Some patients/public also had difficulties in clarifying and/or undertaking their assigned roles/tasks during the review process^35^, ^36^, ^41^ or did not have the opportunity to obtain the necessary individual feedback and support from researchers regarding their roles/tasks in the synthesis/review project.^35^, ^36^

##### Time

Patients/public felt that the synthesis/review projects that they had been involved in had a longer duration than they had anticipated.^35^, ^41^ They felt that they did not have adequate time to prepare, and often had to overwork themselves to read papers and other research‐related documents before meetings.^35^, ^41^ They highlighted the need for realistic deadlines and enough time during the synthesis/review process to guarantee their useful contributions/inputs to these projects.^35^, ^41^

*Instructions came very late together with the 11 papers. An immense amount of papers with too short a deadline. I'm sorry but I had no time to read or prepare before the meeting. To be able to do this well together with our ordinary jobs for at least two months (11 papers!). Thinking needs that time I think*. (Patient research partner 2)^35^

*The length of time the research took was quite long…*.^41^

Additionally, the time‐consuming nature of the synthesis/review projects made some patients/public lose personal interest in the current or future synthesis/review projects.^35^, ^41^

##### Low self‐esteem

Patients/public expressed self‐doubt and queried the ‘real’ impact of their involvement on the synthesis/review project, either wholly^35^, ^36^, ^41^ or in a specific way.^35^, ^36^ They expressed frustrations about their seeming lack of or inadequate knowledge regarding the health topic/intervention being assessed or the synthesis/review process,^35^, ^36^ and whether their views were fully considered by researchers in decision‐making processes.^35^, ^41^

*To be honest, I'm not quite sure. I don't know… if I was useful really, not in a general way but more specific. I think the idea of involving patients in this kind of work is potentially very good, but I had a constant lurking feeling of just being an amateur scientist*. (Patient Research partner 2)^35^

Patients/public were quite worried or anxious about the ways/manner they had expressed their personal opinions and/or concerns about the synthesis/review projects.^35^, ^41^ They also expressed worry about their inability to obtain the necessary feedback from researchers to help inform their inputs in the synthesis/review project and about researchers' actions and attitudes towards them.^35^, ^36^

#### 3.5.2 The perspective of researchers

##### Time

Researchers also felt that they did not have adequate time to develop PPI networks,^37^, ^40^ or to plan and conduct PPI in the ES/SR projects^36^, ^39^, ^41^ Also, researchers reported that their interactions with patients/public were limited to the busy times of the project timetable, and this made it difficult for them to build good relationships and foster trust with patients/public.^36^, ^37^, ^40^

*We had no dedicated team member responsible for maintaining end‐user relationships, and at the busiest times in the project timetable, there was little time to think about end‐user involvement. This limited the opportunities for collaboration, most of the involvement being consultative in nature. It was also not possible for the researchers to develop a good rapport with end‐users as the opportunities for relationship building were limited*.^36^

Furthermore, researchers expressed difficulties in involving patients/public at multiple stages in the synthesis/review process due to the duration of the synthesis/review projects.^37^, ^40^ Researchers felt that involving patients/public in the synthesis/review process was a complex task (especially during the stage of data synthesis) which demanded time.^39^, ^41^

##### Balancing inputs and managing relations

Researchers had difficulties in managing the enthusiasm and unrealistic expectations of patients/public relative to the scope of the synthesis/review project^36^, ^40^, ^41^ as well as balancing the priorities and needs of patients/public and researchers relative to these projects.^36^, ^37^, ^40^, ^41^

*Common to several of the end‐user events, managing expectations and balancing the enthusiasm of end‐users with a realisation of what was achievable within the project scope was difficult. As researchers, we did not always feel comfortable with this and were aware, at times, that our skills in this area may not be adequate*.^36^

Researchers felt that there were limited opportunities for patients/public to influence these projects through their inputs/contributions.^36^, ^41^, ^42^ In some instances, researchers experienced difficulties in managing discussions around sensitive topics which directly or indirectly affected patients/public.^36^, ^40^, ^42^ Additionally, researchers had difficulties in sharing control,^37^, ^40^ and limited opportunities for building good relationships between patients/public and themselves to facilitate cooperation and coordination for mutual benefits.^36^, ^41^

##### Resources and training

Researchers felt that they encountered difficulties in obtaining funding for ES/SR projects which involved patients/public,^37^, ^40^ although such projects were often seen by researchers to be resource‐intensive.^36^, ^37^, ^38^, ^40^, ^41^ They felt that they needed additional funding to ensure that PPI had a meaningful impact on the findings or synthesis/review project.^38^, ^42^

*Finally, we hoped to involve young people at the end of our review to share our results but were unable to do this due to limited resources and time*.^38^

Moreover, researchers acknowledged that they sometimes lacked the requisite skills to properly involve patients/public in and/or effectively manage the synthesis/review process.^36^, ^40^

*…As researchers, we did not always feel comfortable with this [managing expectations and balancing the enthusiasm of end‐users] and were aware, at times, that our skills in this area may not be adequate*.^36^

They also agreed that the lack of or inadequate training and support for patients/public could limit their meaningful involvement in the synthesis/review process.^36^, ^37^, ^40^, ^41^

### 3.6 Recommendations for best practice

#### 3.6.1 Provision of sufficient time and resources

To facilitate meaningful PPI, most authors recommended that adequate time and resources need to be made available for researchers conducting ES/SR projects.^35^, ^36^, ^37^, ^40^, ^41^ Authors argued that researchers need to consider PPI ahead of the project schedule^36^, ^37^, ^40^ and set realistic timelines for their involvement.^40^, ^41^

*Early consideration of PPIE [patient and public involvement and engagement] involvement in order to plan and allocate enough time and funding’* ^40^

*Ensure that you add adequate time and resources to your project for meaningful PPI [patient and public involvement]*.^35^

Authors recommended that researchers need to consider the cost–benefit implications of PPI, seek advice and request adequate funds and other resources to support and sustain the involvement of patients/public in the synthesis/review process.^36^, ^37^, ^40^, ^41^

*Researchers planning to involve patients in their research should request additional resources in funding applications*.^41^

Additionally, authors recommended that researchers must compensate patients/public for their time at the recommended rate as well as reimburse any expenses (such as travel, etc.).^37^, ^40^

#### 3.6.2 Developing a clear recruitment plan

The authors suggested that researchers need to devise a well‐defined plan for PPI.^36^, ^37^, ^40^, ^41^, ^42^ Researchers need to consider ‘who’, ‘when’ and ‘how’ to recruit patients/public—based on their personal interests and expertise—and the appropriate number of PPI to involve in specific projects.^36^, ^37^ The authors suggested that researchers may recruit patients/public from established PPI support networks/groups and utilise an advisory group to offer advice to researchers and facilitate PPI.^37^, ^41^

*Develop a clear plan for end‐user involvement and a central point for recruiting end‐users, allowing sufficient time and resource to allow co‐ordination and maintenance of contact throughout the project period*.^36^

The authors also recommended that there is the need to recruit a dedicated PPI coordinator to liaise between researchers and patients/public.^37^, ^40^, ^42^

*Consider the involvement of a dedicated PPIE [patient and public involvement and engagement] facilitator*.^42^

Additionally, researchers must adopt a flexible approach to PPI and define, clarify and document their level of involvement throughout the synthesis/review project.^36^, ^40^ Some authors agreed that patients/public should be given the opportunity to withdraw from these projects at any time after researchers have explained the nature of their involvement to them.^40^, ^41^

#### 3.6.3 Provision of sufficient training and support

Researchers should identify the basic training and support needs of patients/public before their involvement,^35^, ^37^ develop and share appropriate training materials and other resources with them, and evaluate the impact of training resources and sessions on their involvement. Researchers should provide the necessary feedback to patients/public to facilitate their meaningful contributions to every stage of the synthesis/review process.^35^, ^37^, ^40^

*Discuss members' training needs, recognising individuals will have different experiences. Consider sharing existing training resources*.^37^

*Consider holding pre‐workshops for service users to learn about methods and discuss experiences so that they are more comfortable with ‘experts’ and can rehearse contributions*.^36^

The authors also suggested that patients/public should be provided with different communication avenues (including emails, post, telephone, online and written), with discussions employing either an individual or a focus group format.^35^, ^37^, ^40^, ^42^

*Give members different ways of expressing their opinion and any concerns (written, online, within‐group, individually)*.^37^

*Utilisation of focus group with meaningful number of participants to discuss review findings*.^35^

#### 3.6.4 Need to foster positive working relationships

Researchers must acknowledge the need to manage power imbalances between patients/public and themselves throughout the process, as well as resolve conflicts between patients/public involved.^36^, ^37^, ^40^, ^42^

*Recognise power relations can be an issue to manage. Clearly recognise and appreciate PPIE [patient and public involvement and engagement] members expertise*.^37^

Authors agreed that researchers ought to value the patients/public involved and acknowledge their potential inputs/contributions to the synthesis/review process.^35^, ^37^, ^40^ However, researchers must properly manage the expectations of patients/public and ensure that they are feasible for the project.^36^, ^37^, ^40^, ^42^

Additionally, researchers must liaise with the PPI network(s) to facilitate an effective, ‘two‐way’ communication targeted at creating mutual motivations and enhancing research outputs.^40^, ^42^ Researchers, together with patients/public, must create and consent to a formal ‘code of conduct’ to guide the actions and behaviours of team members during meetings and ensure mutual respect.^36^, ^40^

*Develop and agree a clear ‘ground rules’ for meetings and events which allow the contributions of individuals to be valued and respected*.^36^

## 4 DISCUSSION

This SR synthesised findings from eight papers that assessed the benefits, challenges and best practice(s) for PPI in individual, health‐related ES/SRs projects from the perspectives of two *specific groups of stakeholders*: patients/public and researchers. The *benefits* described from patients'/public's perspective included gaining knowledge about ES/SRs and feeling empowered to shape ES/SR projects, whereas *benefits* from the researchers' perspective were improving the quality of the review processes, enhancing the relevance of outcomes from ES/SRs, and enhancing the dissemination of findings. The *challenges* described from patients'/public's perspective included: poor communication between researchers and patients/public; time constraints in making relevant contributions to synthesis/review projects; and low self‐esteem about patients'/public's impact on ES/SRs. *Challenges* from the researchers' perspective were inadequate resources and training of both researchers and patients/public; time constraints in involving patients/public; and balancing inputs and managing relations between researchers and patients/public. To facilitate meaningful PPI in ES/SRs, authors recommended best practices such as providing adequate time and resources for involvement processes, developing a clear plan for recruiting patients/public, providing adequate training and support for both researchers and patients/public, and building good working relationships between researchers and patients/public.

Our review reports the benefits and challenges of PPI in ES/SRs projects from the perspectives of two *specific groups of stakeholders* (i.e., researchers and patients/public) for the first time. The themes on *benefits* and *challenges* in our review are consistent with findings from other SRs^17^, ^43^, ^44^ that focussed on describing patients'/public's and/or researchers' involvement in other types of research^43^, ^44^ or included the analysis of interview reports from other stakeholders such as programme leaders.^17^ The evidence from our review suggests that the benefits and challenges of PPI in individual ES/SR projects are described differently from the points of view of patients/public and researchers. This may be related to the seemingly divergent opinions, assumptions, needs, priorities, values and/or motivations for patients'/public's and researchers' involvement in these projects. From the perspective of researchers, the *benefits* reported enabled researchers to generate new ideas and develop competencies in conducting PPI in ES/SRs. Thus, enabling researchers to design appropriate recruitment strategies, identify and prioritise relevant data and outcomes in the synthesis/review projects, and increase the uptake of review findings in future ES/SR studies. However, the perspectives of patients/public generally highlighted the personal benefits from their involvement and the significance of patient and community involvement in decisions regarding their health. Thus, these benefits can help promote their sustained involvement in shaping the best evidence‐based health research and its utilisation; assist the development, implementation and evaluation of health services for the public; and improve patient‐centred health outcomes.

The different perspectives shared by patients/public and researchers during involvement processes resulted in significant challenges, which may have the potential to affect research integrity in the synthesis/review process and could result in researchers being less motivated to involve patients/public in ES/SR projects. This may limit opportunities to make these projects more appropriate and relevant for patients/public. Also, these challenges could generate frustration among patients/public and conflicts/tensions between patients/public and researchers, thereby, hampering patients'/public's motivation and response to future involvement in these projects.

The evidence on *best practice* in our review is consistent with findings from another study^45^ which assessed patients' perspective of meaningful engagement in SRs, although this study did not highlight the need to provide training for researchers on the design and implementation of PPI in ES/SR projects. The evidence from our review suggests that researchers need to clearly preplan PPI in ES/SR projects (particular consideration should be given to resources, time and recruitment strategies); provide relevant training and support to both patients/public and researchers; and actively foster trusting relationships between patients/public and researchers. The meaningful involvement of patients/public in research has centred on the provision of adequate training for patients/public. However, the significance of training programmes/workshops for researchers who involve patients/public in other types of research has also been reported,^46^ and this form of training could be essential for researchers involving patients/public in their ES/SR projects. PPI training for researchers has the potential to enhance their skills and competencies in involving patients/public at every stage of the ES/SR process and develop their knowledge and expertise in applying for research funding. PPI training for researchers can also support their utilisation of existing frameworks (e.g., the ACTIVE framework and the GRIPP2 checklist) to aid the design, implementation, and reporting of PPI in ES/SR projects.

### 4.1 Strengths and limitations

#### 4.1.1 Strengths

This review has a number of strengths. It utilised the methods of thematic synthesis by Thomas and Harden,^33^ a widely used procedure in the health sciences which offers a transparent process in synthesising data from qualitative studies with varying reporting styles. Included studies had moderate‐to‐high methodological quality, and the use of the GRADE‐CERQual approach suggests that there is good confidence in the review findings to support its use in decision‐making processes, including policy formulation and guideline development.

#### 4.1.2 Limitations

The review also has some limitations. Firstly, the lack of a common reporting style (and other reporting limitations) posed difficulties in locating and extracting the ‘right’ data from the selected studies. As such, some relevant data may have been missed at the data extraction stage, although data extraction was undertaken by two independent reviewers to minimise this risk. Again, there were difficulties in distinguishing between the evidence from patients'/public's perspectives due to the mixed reporting of these points of view in some of the included primary studies; hence, both groups were treated as ‘patients and the public’^8^ in this review. Additionally, studies published in languages other than English were excluded, and this might lead to language bias. Finally, most included studies were conducted in the United Kingdom, and this limits the generalisability/transferability of findings to other settings, particularly to low‐ and middle‐income countries (LMICs).

### 4.2 Implications for research and practice

#### 4.2.1 Implications for research

Future studies must be targeted at the utilisation of existing frameworks (e.g., ACTIVE framework) by researchers to help describe the approaches and methods for involving patients/public in ES/SRs. Additionally, future studies from diverse settings (including LMICs) should aim to publish the benefits, challenges, context and processes of involving patients/public to help locate essential components which facilitate meaningful PPI in synthesis/review projects (e.g., recruitment strategies, medium of communication, etc.).

#### 4.2.2 Implications for practice

The evidence from this review suggests the need for ES/SR‐related organisations to utilise context‐specific resources/materials on the involvement of patients/public in individual synthesis/review projects, including compensation guidelines and nonpecuniary methods for acknowledging patients'/public's skills and time, budgeting for involvement activities, and so forth. Additionally, guidance on context‐specific training and support for both researchers and patients/public should include who receives what specific type of training, method of training, organising training events, ways to build a good rapport between researchers and patients/public, and so forth.

For organisations undertaking rapid reviews (RRs), the stage and level of involvement of patients/public may depend on the interests and abilities of the patients/public recruited, the project timeline, available funding, and whether patients/public will be involved in a single RR or multiple RRs in a related domain of healthcare.

## 5 CONCLUSION

This qualitative SR provides a comprehensive overview of the benefits, challenges and best practices for PPI in ES/SR projects from the perspectives of both patients/public and researchers. PPI constitutes an essential component to high‐quality research and accentuating the benefits and challenges of PPI from the perspectives of primary stakeholders is essential for meaningful involvement in ES/SRs. Again, PPI in ES/SRs is at the nascent stage and requires careful consideration of approaches and methods for involvement and evaluating impacts from both the patients'/public's and researchers' perspectives.

## AUTHOR CONTRIBUTIONS

Eldad Agyei‐Manu, Jasmin Rostron and Ruth McQuillan made contributions to conception and design of the review. Eldad Agyei‐Manu, Nadege Atkins, Jasmin Rostron, Maureen Smith and Ruth McQuillan contributed to data collection and interpretation. Eldad Agyei‐Manu, Bohee Lee and Marshall Dozier contributed to the search strategy. Eldad Agyei‐Manu wrote the first draft of the manuscript. All authors contributed to the manuscript revision and approval of the final submission.

## CONFLICT OF INTEREST STATEMENT

The authors declare no conflict of interest.

## ETHICS STATEMENT

Ethical approval was obtained from the Usher Masters' Research Ethics Group (UMREG) of the University of Edinburgh, UK (Supporting Information: File S2).

## Supporting information

Supporting information.

Supporting information.

Supporting information.

Supporting information.

Supporting information.

Supporting information.

## Data Availability

Data sharing is not applicable to this article as no new data were created in this study.
