# Supplementary material for: The benefits, challenges, and best practice for patient and public involvement in evidence synthesis: A systematic review and thematic synthesis
Source: Health Expect. 2023 Jun 1;26(4):1436–52. doi: 10.1111/hex.13787 (PMC10349234; doi:10.1111/hex.13787)
Supplement: Supplementary file 1 — Supporting information. [file HEX-26--s002.docx]

**Supplementary File 1: Search strategy**

**Supplementary File 1a. MEDLINE search (Date of search: 02/02/2022)**

***Retrieved records = 1,959 studies***

| **No.** | **Query** |
| --- | --- |
| 1 | ((patient* or stakeholder* or public or user* or community or consumer* or citizen*) adj1 (engag* or involv* or participa*)).ti,ab,kw,kf. |
| 2 | Stakeholder Participation/ |
| 3 | Patient Participation/ |
| 4 | (patient and public involvement).mp. |
| 5 | 1 or 2 or 3 or 4 |
| 6 | (systematic adj (review$1 or overview$1)).tw. |
| 7 | exp "Review Literature as Topic"/ |
| 8 | exp Systematic Reviews as Topic/ |
| 9 | "evidence synthes*".tw. |
| 10 | "evidence-based review*".tw. |
| 11 | guideline.pt. |
| 12 | 6 or 7 or 8 or 9 or 10 or 11 |
| 13 | 5 and 12 |
| 14 | animal/ |
| 15 | human/ |
| 16 | 14 not (14 and 15) |
| 17 | Comment/ |
| 18 | Letter/ |
| 19 | Editorial/ |
| 20 | or/16-19 |
| 21 | 13 not 20 |
| 22 | exp Health Services/ |
| 23 | exp Health Services Research/ |
| 24 | exp Research Design/ |
| 25 | "health service*".mp. |
| 26 | "clinical research*".mp. |
| 27 | "health research*".mp. |
| 28 | "health care*".mp. |
| 29 | "healthcare*".mp. |
| 30 | 22 or 23 or 24 or 25 or 26 or 27 or 28 or 29 |
| 31 | 21 and 30 |

**Supplementary File 1b. EMBASE search (Date of search: 02/02/2022)**

***Retrieved records = 5,638 studies***

| **No.** | **Query** |
| --- | --- |
| 1 | ((patient* or stakeholder* or public or user* or community or consumer* or citizen*) adj1 (engag* or involv* or participa*)).ti,ab,kw,kf. |
| 2 | Stakeholder engagement/ |
| 3 | Patient participation/ |
| 4 | (patient and public involvement).mp. |
| 5 | 1 or 2 or 3 or 4 |
| 6 | (systematic adj (review$1 or overview$1)).tw. |
| 7 | exp Review Literature as Topic/ |
| 8 | "evidence synthes*".tw. |
| 9 | "evidence-based review*".tw. |
| 10 | "systematic review"/ |
| 11 | 6 or 7 or 8 or 9 or 10 |
| 12 | 5 and 11 |
| 13 | animal/ |
| 14 | human/ |
| 15 | 13 not (13 and 14) |
| 16 | Comment/ |
| 17 | Letter/ |
| 18 | Editorial/ |
| 19 | or/15-18 |
| 20 | 12 not 19 |
| 21 | exp health service/ |
| 22 | exp health services research/ |
| 23 | "health service*".mp. |
| 24 | "clinical research*".mp. |
| 25 | "health research*".mp. |
| 26 | "health care*".mp. |
| 27 | "healthcare*".mp. |
| 28 | exp Research Design/ |
| 29 | 21 or 22 or 23 or 24 or 25 or 26 or 27 or 28 |
| 30 | 20 and 29 |

**Supplementary File 1c. CINAHL Plus search (Date of search: 02/02/2022)**

***Retrieved records = 2,163 studies***

| **No.** | **Query** | **Search Options** |
| --- | --- | --- |
| S1 | TI ( ((patient* or stakeholder* or public or user* or community or consumer* or citizen*) N1 (engag* or involv* or participa*)) ) OR AB ( ((patient* or stakeholder* or public or user* or community or consumer* or citizen*) N1 (engag* or involv* or participa*)) ) | Boolean/Phrase |
| S2 | MM "Consumer Participation" | Find all my search terms |
| S3 | MM “Stakeholder Participation” | Find all my search terms |
| S4 | TX “patient and public involvement” | Find all my search terms |
| S5 | S1 OR S2 OR S3 OR S4 | Find all my search terms |
| S6 | MM "systematic review" | Find all my search terms |
| S7 | TX "systematic review*" | Find all my search terms |
| S8 | TX "evidence-based review*" | Find all my search terms |
| S9 | TX "evidence synthes*" | Find all my search terms |
| S10 | S6 OR S7 OR S8 OR S9 | Find all my search terms |
| S11 | S5 AND S10 | Find all my search terms |
| S12 | Animal/ | Find all my search terms |
| S13 | Human/ | Find all my search terms |
| S14 | S12 NOT (S12 AND S13) | Find all my search terms |
| S15 | Comment/ | Find all my search terms |
| S16 | Letter/ | Find all my search terms |
| S17 | Editorial/ | Find all my search terms |
| S18 | S14 OR S15 OR S16 OR S17 | Find all my search terms |
| S19 | S11 NOT S18 | Find all my search terms |
| S20 | MM “Health Services+” | Find all my search terms |
| S21 | MM “Health Services Research+” | Find all my search terms |
| S22 | TX "health service*" | Find all my search terms |
| S23 | TX "clinical research*" | Find all my search terms |
| S24 | TX "health research*" | Find all my search terms |
| S25 | TX "health care*" | Find all my search terms |
| S26 | TX "healthcare*" | Find all my search terms |
| S27 | S20 OR S21 OR S22 OR S23 OR S24 OR S25 OR S26 | Find all my search terms |
| S28 | S19 AND S27 | Find all my search terms |

**Supplementary File 1d. Cochrane Library search (Date of search: 02/02/2022)**

***Retrieved records = 135 studies***

| **No.** | **Query** |
| --- | --- |
| 1 | (((patient* or stakeholder* or public or user* or community or consumer* or citizen*) NEAR/1 (engag* or involv* or participa*))):ti,ab,kw. |
| 2 | MeSH descriptor Patient Participation, this term only |
| 3 | MeSH descriptor Stakeholder Participation, this term only |
| 4 | #1 OR #2 OR #3 |
| 5 | Limit 4 to “Cochrane Reviews” |
