# Supplementary figures and images for: The benefits, challenges, and best practice for patient and public involvement in evidence synthesis: A systematic review and thematic synthesis
Source: Health Expect. 2023 Jun 1;26(4):1436–52. doi: 10.1111/hex.13787 (PMC10349234; doi:10.1111/hex.13787)

**Supplementary File 2: Level 1 ethics oversight letter from UMREG**
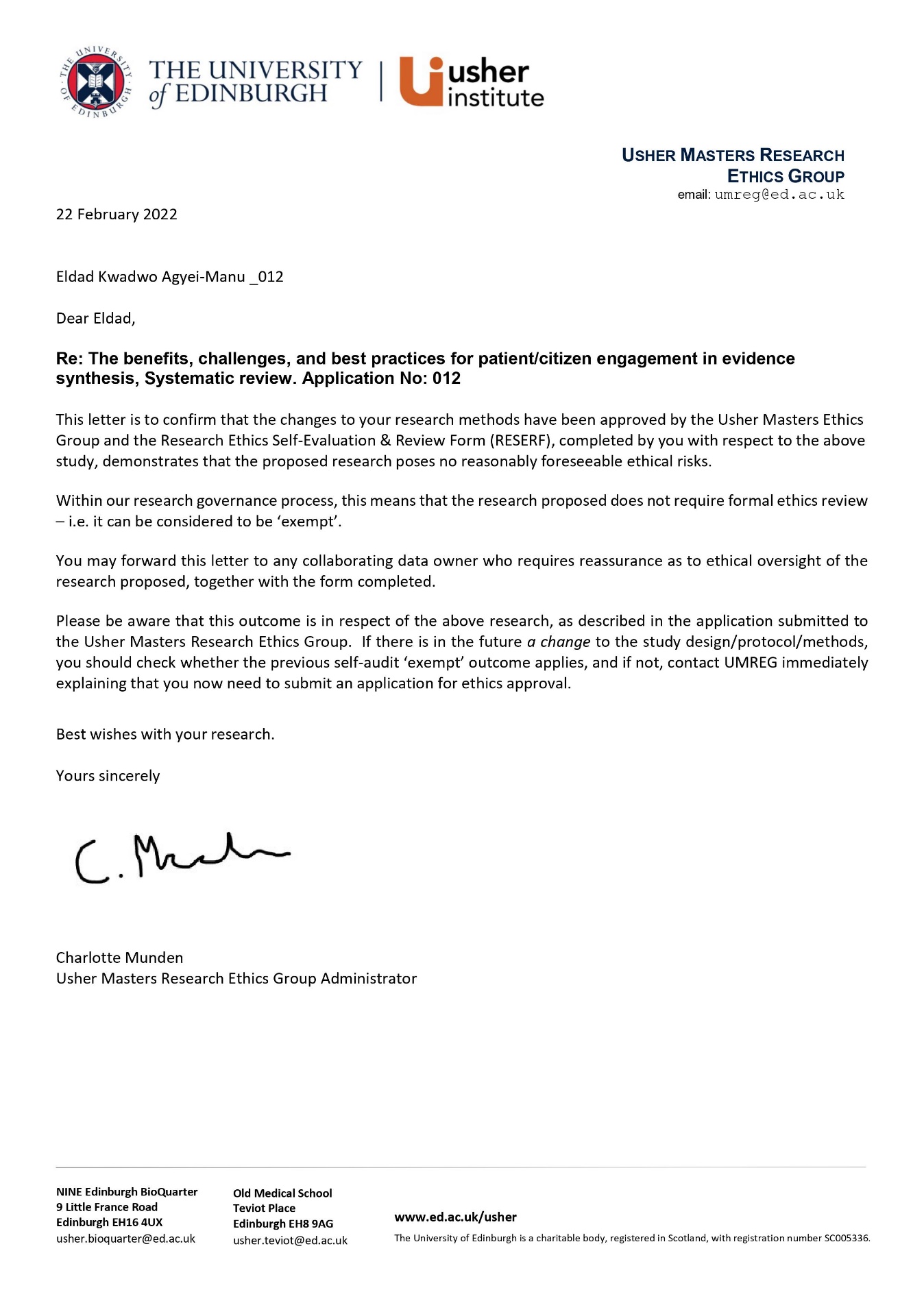

Supplement: Supplementary file 2 — Supporting information. [file HEX-26--s004.docx]
