# Supplementary material for: The benefits, challenges, and best practice for patient and public involvement in evidence synthesis: A systematic review and thematic synthesis
Source: Health Expect. 2023 Jun 1;26(4):1436–52. doi: 10.1111/hex.13787 (PMC10349234; doi:10.1111/hex.13787)
Supplement: Supplementary file 3 — Supporting information. [file HEX-26--s003.docx]

**Supplementary File 3: Summary on Qualit****ative evidence profile using the GRADE-CERQual approach**

| **Finding** | **Studies Supporting the Finding** | **Evaluation of Methodological Limitations** | **Evaluation of Relevance** | **Evaluation of Coherence** | **Evaluation of Adequacy** | **General Evaluation of Confidence** | **Reasons for Decision** |
| --- | --- | --- | --- | --- | --- | --- | --- |
| **BENEFITS** | | | | | | | |
| Gaining knowledge | ^34,40,41^ | Small issues pertaining to methodological limitations (three studies with slight methodological limitation) | Small issues pertaining to relevance (studies of process and impact of PPI in SRs, having appropriate data from four European countries: UK, Romania, Sweden, and Estonia) | Small issues pertaining to coherence (consistency in data across and within studies) | Moderate issues pertaining to adequacy (three studies generally presented low-to-moderate rich data) | Moderate | Small issues with reference to relevance, methodological limitations, and coherence. |
| Empowerment | ^34,35,40,41^ | Small issues pertaining to methodological limitations (three studies having slight issues and one study with moderate methodological limitation) | Small issues pertaining to relevance (studies of process and impact of PPI in SRs, having appropriate data from four European countries: UK, Romania, Sweden, and Estonia) | Small issues pertaining to coherence (consistency in data across and within studies) | Small issues pertaining to adequacy (four studies generally presented moderate rich data) | Moderate | Small issues with reference to relevance, methodological limitations, adequacy, and coherence. |
| Improving quality | ^35-41^ | Small issues pertaining to methodological limitations (five studies having slight issues and two studies with moderate methodological limitation) | Small issues pertaining to relevance (studies of process and impact of PPI in SRs, having appropriate data from one European country, UK) | Small issues pertaining to coherence (consistency in data across and within studies) | Small issues pertaining to adequacy (seven studies generally presented moderate rich data) | Moderate | Small issues with reference to relevance, methodological limitations, adequacy, and coherence. |
| Enhancing relevance | ^35-41^ | Small issues pertaining to methodological limitations (five studies having slight issues and two studies with moderate methodological limitation) | Small issues pertaining to relevance (studies of process and impact of PPI in SRs, having appropriate data from one European country, UK) | Small issues pertaining to coherence (consistency in data across and within studies) | Small issues pertaining to adequacy (seven studies generated presented moderate rich data) | Moderate | Small issues with reference to relevance, methodological limitations, adequacy, and coherence. |
| Enhancing dissemination of findings | ^36,39-41^ | Small issues pertaining to methodological limitations (three studies having slight issues and one study with moderate methodological limitation) | Small issues pertaining to relevance (studies of process and impact of PPI in SRs, having appropriate data from one European country, UK) | Small issues pertaining to coherence (consistency in data across and within studies) | Moderate issues pertaining to adequacy (four studies generally presented low-to-moderate rich data) | Moderate | Small issues with reference to relevance, methodological limitations, adequacy, and coherence. |
| **CHALLENGES** | | | | | | | |
| Poor communication | ^34,35,40^ | Small issues pertaining to methodological limitations (two studies having slight issues and one study with moderate methodological limitation) | Small issues pertaining to relevance (studies of process and impact of PPI in SRs, having appropriate data from four European countries: UK, Romania, Sweden, and Estonia) | Small issues pertaining to coherence (consistency in data across and within studies) | Small issues pertaining to adequacy (three studies generally presented moderate rich data) | Moderate | Small issues with reference to relevance, methodological limitations, and coherence. |
| Time | ^34-36,38-40^ | Small issues pertaining to methodological limitations (four studies having slight issues and two studies with moderate methodological limitation) | Small issues pertaining to relevance (studies of process and impact of PPI in SRs, having appropriate data from four European countries: UK, Romania, Sweden, and Estonia) | Small issues pertaining to coherence (consistency in data across and within studies) | Small issues pertaining to adequacy (six studies generally presented moderate rich data) | Moderate | Small issues with reference to relevance, methodological limitations, adequacy, and coherence. |
| Low self-esteem | ^34,35,40^ | Small issues pertaining to methodological limitations (two studies having slight issues and one study with moderate methodological limitation) | Small issues pertaining to relevance (studies of process and impact of PPI in SRs, having appropriate data from four European countries: UK, Romania, Sweden, and Estonia) | Small issues pertaining to coherence (consistency in data across and within studies) | Small issues pertaining to adequacy (three studies generally presented moderate rich data) | Moderate | Small issues with reference to relevance, methodological limitations, adequacy, and coherence. |
| Balancing inputs and managing relations | ^35,36,39-41^ | Small issues pertaining to methodological limitations (three studies having slight issues and two studies with moderate methodological limitation) | Small issues pertaining to relevance (studies of process and impact of PPI in SRs, having appropriate data from one European country, UK) | Small issues pertaining to coherence (consistency in data across and within studies) | Small issues pertaining to adequacy (seven studies generally presented moderate rich data) | Moderate | Small issues with reference to relevance, methodological limitations, adequacy, and coherence. |
| Resources and training | ^35-37,39-41^ | Small issues pertaining to methodological limitations (four studies having slight issues and two studies with moderate methodological limitation) | Small issues pertaining to relevance (studies of process and impact of PPI in SRs, having appropriate data from one European country, UK) | Small issues pertaining to coherence (consistency in data across and within studies) | Small issues pertaining to adequacy (six studies generally presented moderate rich data) | Moderate | Small issues with reference to relevance, methodological limitations, adequacy, and coherence. |
| **RECOMMENDATIONS FOR BEST PRACTICE** | | | | | | | |
| Provision of sufficient time and resources | ^34-36,39,40^ | Small issues pertaining to methodological limitations (three studies with slight issues and two studies with moderate methodological limitation) | Small issues pertaining to relevance (studies of process and impact of PPI in SRs, having appropriate data from four European countries: UK, Romania, Sweden, and Estonia) | Small issues pertaining to coherence (consistency in data across and within studies) | Small issues pertaining to adequacy (five studies generally presented moderate rich data) | Moderate | Small issues with reference to relevance, methodological limitations, adequacy, and coherence. |
| Developing a clear recruitment plan | ^35,36,39-41^ | Small issues pertaining to methodological limitations (three studies having slight issues and two studies with moderate methodological limitation) | Small issues pertaining to relevance (studies of process and impact of PPI in SRs, having appropriate data from one European country, UK) | Small issues pertaining to coherence (consistency in data across and within studies) | Small issues pertaining to adequacy (five studies generally presented moderate rich data) | Moderate | Small issues with reference to relevance, methodological limitations, adequacy, and coherence. |
| Provision of sufficient training and support | ^34-36,39-41^ | Small issues pertaining to methodological limitations (four studies having slight issues and two studies with moderate methodological limitation) | Small issues pertaining to relevance (studies of process and impact of PPI in SRs, having appropriate data from four European countries: UK, Romania, Sweden, and Estonia) | Small issues pertaining to coherence (consistency in data across and within studies) | Small issues pertaining to adequacy (six studies generally presented moderate rich data) | Moderate | Small issues with reference to relevance, methodological limitations, adequacy, and coherence. |
| Need to foster positive working relationships | ^34-36,39,41^ | Small issues pertaining to methodological limitations (three studies having slight issues and two studies with moderate methodological limitation) | Small issues pertaining to relevance (studies of process and impact of PPI in SRs, having appropriate data from four European countries: UK, Romania, Sweden, and Estonia) | Small issues pertaining to coherence (consistency in data across and within studies) | Small issues pertaining to adequacy (five studies generally presented moderate rich data) | Moderate | Small issues with reference to relevance, methodological limitations, adequacy, and coherence. |
