# Supplementary material for: The benefits, challenges, and best practice for patient and public involvement in evidence synthesis: A systematic review and thematic synthesis
Source: Health Expect. 2023 Jun 1;26(4):1436–52. doi: 10.1111/hex.13787 (PMC10349234; doi:10.1111/hex.13787)
Supplement: Supplementary file 4 — Supporting information. [file HEX-26--s005.docx]

**Supplementary File 4: Description of the benefits and challenges of PPI in evidence synthesis/systematic review projects from the perspectives of patients/public and researchers**

| **No** | **Author**  **(year)** | **Benefits described** | | **Challenges described** | | |
| --- | --- | --- | --- | --- | --- | --- |
|  |  | **Patients’/public’s perspective** | **Researchers’ perspective** | **Patients’/public’s perspective** | **Researchers’ perspective** |  |
| 1 | Bayliss et al., 2016 | - “The PRPs in this study reported that their involvement had personal benefits which included building new skills, improving confidence, and gaining knowledge”.   “*Taking into consideration that it was the first activity of that kind in my experience as a Patient Research Partner, I was very happy and thankful to gain experience in coding. I improved my skills in summarizing, finding key words and interpreting. I gained confidence in participating to future coding activities. It was also very interesting to see how other people think about RA and prevention – very educative (Patient research partner 1)*”  “*I feel I am contributing and being nosey; I get to find things out (Patient research partner 3)*”  “*[Benefits included the] sharing of ideas and experiences (Patient research partner 6)*” | N/A | - “One PRP questioned the real benefits of their contribution to qualitative meta-synthesis”.   “*To be honest, I’m not quite sure. I don’t know… if I was useful really, not in a general way but more specific. I think the idea of involving patients in this kind of work is potentially very good, but I had a constant lurking feeling of just being an amateur scientist (Patient Research partner 2)*”   - “One PRP stated that some of the language used in guidance materials was not clear… Patients stated that they needed more support in understanding the terminology in the papers on these other conditions, especially when English was not their primary language”.   “*Yes, some simplified explanations of the relevance and use of ‘themes’, ‘sub-themes’ and ‘coding’ would have been useful. An understanding of ‘a qualitative literature synthesis’ is not a given (Patient research partner 4)*.”  “*It was very difficult to comprehend fully research documents dealing with conditions other than RMDs [Rheumatic and Musculoskeletal Diseases] without an appropriate glossary etc. (Patient research partner 4)*”   - “Although patients were instructed to focus on the results section of the papers, there was still some confusion on which part of the article was most relevant to the task”.   “*Maybe a talk with the researcher and the other patient partners (so that we can raise questions). Also, some training on how to read an article (for example, is it better to focus more on the Abstract, Discussion and Conclusion or is it better to read carefully the whole document in order not to miss important issues/ideas?) (Patient research partner 1)*”  “*Perhaps face to face (training) to explain the process and to answer questions [would be better] (Patient research partner 3)*”   - “Only one individual believed that they had enough time to complete this activity [of coding three papers]. The other patients believed that this was not enough time for them to work through the papers when they had other family and work commitments”.   “*Instructions came very late together with the 11 papers. An immense amount of papers with too short a deadline. I’m sorry but I had no time to read or prepare before the meeting. To be able to do this well together with our ordinary jobs at least two months (11 papers!). Thinking needs that time I think (Patient research partner 2)*”  “*Sending 11 documents for reading before a meeting the following week was unrealistic, for me at least. In fact, there was so much data that my inbox would not accept it (Patient research partner 4)*”  “*No, I didn’t [have enough time]. I remember I was working the whole duration of the flight. Taking into consideration that there were many articles, I would have liked to have two-three weeks at my disposal (this is also because of my part-time job and the fact that sometimes I get to work only at weekends) (Patient research partner 1)*”   - “PRPs highlighted that they would have liked more detailed feedback including whether any improvements could be made”.   “*A quick feedback after paper 1 to make sure I was doing what you wanted, with further requests from the researchers if needed. We PRP’s want to help the best we can (Patient research partner 2)*”  “*I would have liked some quick feedback… to get a notion of whether I could improve anything, whether I had understood the instructions (Patient research partner 2)*”  “*I would have liked to have another call with the researcher [to] explain to me what was good and what was wrong and give me examples/ideas on how to improve what was wrong (Patient research partner 1)*”   - “The evaluation for this project was conducted 15 months after the event due to the researcher going on maternity leave. PRPs stated that this gap meant that they sometimes struggled to remember the details of taking part in the study”.   “*For the future it would be very helpful if we can fill in the evaluation forms in one or two months after completing the activity (Patient research partner 1)*” | N/A |  |
| 2 | Coon et al., 2016 | - “There was recognition that previous experience with end-user involvement is likely to inform future attitude. Open exploration of the beliefs and perceptions of individuals on the project team about the potential benefits and costs of end-user involvement at the start of the project was found to be beneficial” | - “The event [which was organized to share information about project and to explore end-user knowledge and experience] resulted in the identification of over 40 nonpharmacological interventions for ADHD used in schools… and were used to inform the search strategies for the reviews. Over 40 outcomes that could be used to evaluate the effectiveness of interventions were also identified. A typology of interventions and outcomes was produced and used to inform the shape of the reviews” - “Participants confirmed the relevance of the interim themes of the reviews in recognizing the issues and confirming many as important, and this established the potential for transferability of the interim findings. Participants were also able to offer commentary and critique to the themes, which supported the direction of the continuing analysis” - “The opportunity to share preliminary findings with end-users and then to revisit both the synthesis and the included papers was welcomed, a process which provided a sense of confidence and belief in the findings” | - “Reflection from one of the contributors [end-user], however, highlighted that although this was an interesting meeting [to discuss the emerging findings from the reviews] and they were pleased to help, because we [researchers] were unable to give them clear guidance on which interventions they should be using (or not using), they felt that the meeting had limited benefit for them” - “It was also apparent from the reflections of contributors [end-users] to Event 2 that although they were happy to help, because the results were not clear-cut, the engagement was not as fruitful for them as it might have been” - “CS [end-user] reflected that reading the draft chapters had been an emotional experience for her. ADHD affects many aspects of her life, with several members of her family having an ADHD diagnosis; reading about the difficulties that people with ADHD face in black and white reminded her of the costs of ADHD to her family and was painful” | - “Common to several of the end-user events, managing expectations and balancing the enthusiasm of end-users with a realization of what was achievable within the project scope was difficult. As researchers, we did not always feel comfortable with this and were aware, at times, that our skills in this area may not be adequate” - “Managing the intensity of emotion between individuals with differing viewpoints was challenging at times. In particular, there was conflict surrounding the relative importance of various outcomes to different end-users and a suggestion of ‘blame’ both from parents and from teachers” - “Discussing elements of the end-user involvement with both WP and CS [end-users] highlighted the need to balance the relationship so that all parties consider it to be beneficial. As researchers, especially those with a tight deadline, it may be easy to extract what is needed for the project from a group of end-users without carefully considering whether the engagement is mutually beneficial” - “As a group of predominantly methodological researchers, the core team had little prior expertise in communicating with end-users in an evidence synthesis project. Explicit training and time to develop an on-going relationship with end-users would have increased the teams’ confidence in dealing with the challenges. Bridging the gaps in communication between developing and submitting an application for funding and the eventual commencement of the work is an area that we did not manage well. Recruitment of study-specific team members who were not involved in the development of the funding bid meant that there was no on-going relationship between the core research team members and the end-users at the start of the project” - “We involved different groups of end-users throughout the project rather than having one central group who were called on repeatedly. We had no dedicated team member responsible for maintaining end-user relationships, and at the most busy times in the project timetable, there was little time to think about end-user involvement. This limited the opportunities for collaboration, most of the involvement being consultative in nature. It was also not possible for the researchers to develop a good rapport with end-users as the opportunities for relationship building were limited. This highlights the time and resources necessary for meaningful involvement both for the project team and the end-users, and whilst we aimed to achieve a balance between people feeling involved and burdened by the involvement, this might have reduced the sense of being involved for some people” |  |
| 3 | Hyde et al., 2017 | N/A | - “Potential importance of [the] review question to patients [was] established. Clear aims for and design of RUG involvement was established. This facilitated application for funding to support review and patient involvement” - “Importance of review question to patients [was] established. Additional factors of importance [were] identified, and [the] review protocol and data extraction forms [were] amended to reflect these [factors]” - “[There was] reassurance that RUG members raised ideas around relevance of literature that were similar to [that of] the researchers. [There was] reassurance that patient priorities had been reflected in review process. Categories [which were] identified by RUG members were used as framework for the narrative synthesis” - “Results were targeted at practitioners, as RUG members felt [that] this was most important. RUG members and support team participated in dissemination of the review findings. [The] next stage of the research [was] informed by patient perspective and priorities”. | N/A | - “Time pressure (developing PPIE network, building trust, allowing for involvement at more than one time-point)” - “Resources (funding and time)” - “Continuity” - “Concerns about group dynamics (power balance between members and researchers or within the group)” - “Research Ethics Committee involvement” - “‘Representativeness’ of members involved” |  |
| 4 | Jamal et al., 2015 | N/A | - “We found that young people’s views of what to prioritize or how to conceptualize the school environment were consistent with the adult advisory group, the theoretical literature and our own research interests. This congruence provided us with the confidence and rationale to make decisions in our review” - “Young people’s views were also valuable in flagging potential gaps in the evidence. For example, in the first consultation group members identified ‘physical appearance’ or ‘self-image’ as a health concern” - “Group members at both consultations also pointed out that different aspects of health and well-being are interlinked… This helped inform how we approached our synthesis” - “In addition to informing decisions in the review, the consultations helped ensure that the professional research perspective was not the only one brought to bear on interpreting the literature” - “The first consultation with the young people’s advisory group identified how wider societal structures such as social class impact on school health” | N/A | - “Online consultations were not a successful engagement medium. Only two group members registered on the website and only one posted their views (consistent with that reported in face-to-face consultations)” - “It would have been valuable to also illicit views from children to reflect a broader age range, but due to limited resources this was not possible” - “Finally, we hoped to involve young people at the end of our review to share our results but were unable to do this due to limited resources and time”. |  |
| 5 | Oliver et al., 2015 | N/A | - “Reviewers had greater confidence in synthesis findings that had been checked for credibility by a group likely to have perspectives similar to the group under study” - “One of the review’s three implications pertained to gaps in the evidence-base that otherwise might not have been identified by the review team” | N/A | - “The second part [of the views review] did not work as well, as described previously, and this aspect of the PEAR group’s work made no identifiable contribution. Factors limiting this success were a lack of time and a mismatch between the tasks and the nature of the review” - “The production of a qualitative synthesis is a complex task requiring immersion in the data, which was just not possible in the time available” |  |
| 6 | Troya et al., 2019 | N/A | - “Involvement and engagement with PPIE in the research process con‐ tributed to improving relevance, legitimacy, and validity of findings” - “Collaboration and ongoing consultation with PPIE in the research process contributed to the added perspective and understanding of study findings, as well as ensuring a broader capture and prioritization of the public's needs” - “When conducted with adequate support and guidance, PPIE can offer researchers, patients, and the public continuity in the research process. Such was the case when conducting this doctoral research project, given the repeated engagement from PPIE members throughout the study” - “The [PPIE] group also identified gaps in the literature from the SR which they considered as important for patients and public and which require further research” | N/A | - “Time-related pressures” - “Resources (time and funding)” - “Avoiding tokenistic involvement” - “Continuity” - “Clear and open communication” - “Working sensitively” - “Recognition of public involvement” - “More specifically, in doctoral studies, two key challenges for meaningful PPIE involvement are highlighted. Firstly, resources for doctoral research projects. Many doctoral studies are unfunded and/or do not have funding allocated for PPIE, unlike other research projects. This may result in an added difficulty in meaningful involvement of PPIE in doctoral studies. Second, expertise is required for successful PPIE. Doctoral students are often novice researchers, which may require access to expert advice on how to best work sensitively with PPIE members’ needs, including identifying strategies for adequate and tailored support and training, for developing trust and inclusivity” |  |
| 7 | Vale et al., 2012 | - “The Patient Research Partners commented that “*being able to provide some information about late effects and pulling together our thoughts on these issues and getting both in print has been a great achievement” and that, “the outcome passed my expectations, as I witnessed how the team doing the evaluation realized what information was missing and how future trials could give more detailed and long-term information””.* - “The Patient Research Partners all agreed that their main impact on the research was in adding a viewpoint that otherwise would not have been heard and reflecting the concerns of women with cervical cancer by contributing the opinions of “*the normal woman off the street.*” They also felt that their involvement might help to bring about changes, such that “*hopefully future trials will better collect data*” on late side effects” - “All of the Patient Research Partners said they would consider getting involved in research again in the future. “*It was my first experience of user involvement and I would certainly do it again if I felt that my experience was relevant and could usefully contribute to research*.”” | - “Perhaps the greatest impact on the researchers of working with the Patient Research Partners was that it directly led the researchers to get involved in another research project with a greater focus on late side effects of treatment. It also motivated them to publish an editorial with the Patient Research Partners, discussing the concerns of women after treatment for cervical cancer” - “The Patient Research Partners had also helped the researchers to better understand cervical cancer and its treatment, using their experience “*to bring the results of the study to life as it evidenced the experience of real people.*” The researchers felt the greatest impact of involving the Patient Research Partners was the insight they gained of the impact of cervical cancer, its treatments and side effects, on the women’s day to day lives, which would not have been possible without the Patient Research Partner involvement” - “Because the [PPIE] group was already established and used to working together, it had been very easy to involve them in the subsequent work around the drafting of the editorial” | - “One Patient Research Partner did express concerns about her involvement though, *“For a meta-analysis where the outcome measures have already been collected, I am not sure how much difference we have really made overall””* - “One concern raised by the Patient Research Partners was the length of time the project had taken. “*The length of time the research took was quite long, however I understood that collating the information was not an easy task, particularly when trying to locate trials in other countries and getting the information from them*”. One of the key issues this raised was that as time passed, understandably, one of the Patient Research Partners said that her “*personal interest in cancer has faded somewhat.*”” - “Some comments and discussions at the Collaborators’ meeting had been difficult or upsetting for the Patient Research Partners, “*In the lead up to the Collaborators meeting it would have been good to have been better informed about the sorts of discussions that would take place… that the clinicians are going to be blunt and scientific in their approaches and not the normal ’bedside manner’ we might be used to as patients!*”” - “The Patient Research Partners felt that there had been insufficient preparation regarding what the results of the meta-analysis would look like” - “Interestingly, one of the Patient Research Partners expressed that she had concerns at the outset regarding how much input ‘users’ could really make in the context of a systematic review, given that the outcomes are to a degree “*pre-set by the outcomes that were collected within the individual trials*”” | - “In this study, the protocol development took place prior to Patient Research Partners becoming involved, so it was felt that there would be more opportunity for input if Patient Research Partners had been involved earlier in the project”. - “Although the review aimed to evaluate treatment side effects, it was not possible as suitable data were not collected in all of the trials. One of the researchers expressed that this had been disappointing, because it “*felt like we’d perhaps got the Research Partners involved under false pretenses and worried about what they would get out of it because of the lack of data. With hindsight we were perhaps overly optimistic about what we could do.*”” - “For this project, funding received through the UK Department of Health (DoH) NCCRCD Evidence Synthesis award scheme provided the resources to hold meetings, pay the Research Partners for their time and reimburse expenses. The researchers felt that this was important as involving the Patient Research Partners had taken extra time and effort” - “The pressure on researchers’ time only became an issue around key points of the meta-analysis, “*It was sometimes difficult to bridge the needs and priorities of the Patient Research Partners and the clinical/scientific collaborators.*”” - “The researchers felt that they could only commit to a similar approach to patient involvement for systematic reviews based on IPD where there is resource and perhaps as importantly, time, to train, support and develop working relationships with patients. Other potentially less resource-intensive models to involvement could be adopted for standard systematic reviews” |  |
| 8 | Walker et al., 2021 | - “The young people said that in addition to finding their involvement in shaping the research project empowering, they also benefited simply from meeting other CYP with long-term physical conditions and mental health issues. Furthermore, feedback received via email following the first meeting included”:   “*It was good to hear other people's points of view [CYPAG member 1]*”  “*I think it was really helpful having other people who have gone through the same things as you, that understand you [CYPAG member 2]”*  “Parents supported this observation, indicating that they felt it was important for their child to meet other people in similar circumstances to them, who were getting ‘on with their lives’”   - “CYP felt that they would use the knowledge they had gained about the research process in the future”. | - “The contribution of the CYP and their parents enabled us [researchers] to produce a robust evidence synthesis grounded in the experiences and insights of CYP and their parents” - “Our [researchers’] implications for future research were informed not only by existing evidence but also by gaps in the evidence identified by those with experience and insight of the key issues”. - “The meetings [with CYPs] were always felt to be worthwhile in terms of project progress. The researchers found working alongside the CYP both humbling and inspiring, especially given the nature of their physical long-term conditions and mental ill health experiences – life and activities of daily living were very hard for many of the CYP. These were bright, intelligent young people who had experienced significant adversity at young ages. It was motivating, and provoked continual reflection on the needs of the group and how to best engage and involve them in the research process in a way which was truly meaningful and provided an additional sense of accountability to ensure that we conducted the review to the best of our ability”. - “CYP were asked about the terms they use to describe mental health with their peers, to check and make sure the researchers had not missed any key terms for the search strategies of the qualitative and quantitative systematic reviews. This meeting provided the opportunity for researchers to ‘sense-check’ the proposed project”. - “Discussions at this meeting underlined the importance of considering a wide range of outcomes in the reviews including elements like impacts on relationships with friends and family members… These outcomes were not frequently reported in the included studies, and this influenced our recommendations for future research. The researchers hoped the discussion around outcomes might facilitate the structure of the synthesis within the review of quantitative evidence”. - “The discussions provided support to the preliminary themes which were arising from the synthesis of qualitative research and the overarching synthesis… Overall, the discussions with parents and CYP strengthened both the content and final structure of the syntheses conducted within the second and third components of the project”. - “CYP and their parents recorded audio for the podcast and helped to edit plain language summaries. They had strong views on the type of clinicians, educators and researchers the research findings should be shared with, which was incorporated into our dissemination strategy”. - “By continuing our engagement with CYP and their parents through the dissemination stage of our project, we were able to ensure that the messages arising from our project that they felt were most important were communicated to the audiences they felt were most appropriate, via a variety of accessible media” | N/A | - “Planning for meaningful involvement of CYP within this research raised a number of challenges:   (a) we [researchers] needed to involve CYP and accommodate their physical and mental health needs,  (b) we [researchers] were asking CYP to talk about the potentially sensitive topics of their experiences of mental and physical ill health,  (c) the project was a fully funded evidence synthesis with potentially limited opportunities for the involvement to have an impact on findings, and  (d) we [researchers] had a defined budget to cover involvement activities, which would preclude major changes to approach” |  |

Abbreviation: PRP, patient research partner; EuroTEAM, Towards Early biomarkers in Arthritis Management; PARE, People with Arthritis/Rheumatism in Europe; RA, rheumatoid arthritis; PPI, patient and public involvement; ADHD, attention deficit hyperactivity disorder; RUG, research user group; PPIE, patient and public involvement and engagement; ALPHA, Advice Leading to Public Health Advancement; NCB, National Children’s Bureau; PEAR, ‘Public health, Education, Awareness, Research’; CYP, children and young people; LTC, long-term condition; CYPAG, Children and Young People’s Advisory Group
