# Supplementary material for: The benefits, challenges, and best practice for patient and public involvement in evidence synthesis: A systematic review and thematic synthesis
Source: Health Expect. 2023 Jun 1;26(4):1436–52. doi: 10.1111/hex.13787 (PMC10349234; doi:10.1111/hex.13787)
Supplement: Supplementary file 5 — Supporting information. [file HEX-26--s001.docx]

**Supplementary File 5: Authors’ description of best practice for PPI in evidence synthesis/systematic review projects**

| **No.** | **Author**  **(year)** | **Examples of best practice(s) identified** | **Authors’ reasons for being a best practice** | **Authors’ recommendations for best practice** | **Authors’ description of training and support for patients/public involved** | **Timescale for PPI** |
| --- | --- | --- | --- | --- | --- | --- |
| 1 | Bayliss et al., 2016 | 1. Recruiting experienced PRPs from recognized patient groups (e.g., PARE board members) and service user groups 2. Developing and organizing training for PRPs via written materials 3. Obtaining feedback from PRPs during discussions | 1. “To give academic and industry partners feedback on research and provide an insight into the experiences of RA patients” 2. “To promote consistency in techniques used [for data analysis]” 3. “To support the researchers’ coding framework, to support the themes presented by the researchers and highlight the need to report clear recommendations for practice” | - “Provision of adequate training/support to PRPs” - “Provision of adequate time and resources for PPI projects” - “Identifying individual training and support needs before commencement of project” - “Clarification on reasons and levels of PPI involvement in the review” - “Asking PRPs about their time commitment to the project” - “Developing and obtaining feedback from PRPs about training materials” - “Ensuring proper medium of communication between researchers and PRPs (e.g., face-to-face, online)” - “Evaluation of training sessions via questions to PRPs” - “Utilization of focus group with meaningful number of participants to discuss review findings” - “Highlight value of PRP inputs to the review” - “Monitoring and evaluation of PRPs contributions during and after review project” | - “PRPs attended a PRP launch event as part of the wider EuroTEAM study, where they were made aware of what their role as PRP would entail, and the nature of the research they were taking part in”. - “Researchers developed training that was tailored to the roles of patients, in the form of written instructions on how to code the results sections” - “Training material included examples of qualitative research and meta-syntheses techniques, was shortened, and had graphic representations”. - “The first draft of materials was shown to one PRP, who highlighted any sections that were unclear or ambiguous, and the authors made the necessary changes before circulating them”. - “PRPs were given the option within an email of a phone call to talk through the guidance if required”. - “A training document on how to identify themes were sent via email to the PRPs, with the option of phone support”. | - All PRPs were given three (3) weeks to complete their task (i.e., coding of results) - All PRPs were given two (2) weeks to read eleven papers and complete task on identifying themes |
| 2 | Coon et al., 2016 | 1. Recruiting experienced individuals from service user groups and recognized professions 2. Researcher team providing informal training and support to end-users, and vice versa | 1. “To explore their multiple perspectives of lived experience of ADHD in the school setting to enhance the project” 2. “To respond to questions from end-users, and end-users teaching researchers about aspects of ADHD and schools” | - “Encourage open exploration of the views and perceptions of the project team towards the benefits and costs of end-user involvement in the systematic review at the outset of the project”. - “Consider the timing of end-user engagement carefully and schedule meetings/events when they are most likely to have a meaningful impact on the project” - “Develop a clear plan for end-user involvement and a central point for recruiting end-users, allowing sufficient time and resource to allow co-ordination and maintenance of contact throughout the project period” - “Develop and agree a clear ‘ground rules’ for meetings and events which allow the contributions of individuals to be valued and respected”. - “Be clear about the potential for impact of end-user involvement on the people involved and the findings of the systematic review to enable appropriate management of expectations”. - “Consider who to approach as end-users to ensure a breadth of practice, views and perspectives are covered. Take note of the potential for attendees to have shared experiences to ensure that people can feel comfortable talking (e.g., Are the teachers and parents from the same schools? Has the psychiatrist worked with any of the families present?)” - “Allow for flexibility in approach depending on the review topic, the findings, and the clarity of the key messages”. - “Allocate sufficient time and resources to allow for meaningful involvement throughout the project and include end-user involvement processes within the project timetable” - “Consider targeting the involvement and consulting with different end-users for different tasks/aspect of the project depending on their suitability/interests, although this needs to be balance with ensuring that all parties are benefiting from the process”. - “Consider the potential for conflict between end-users with different perspectives when organizing involvement events” - “Consider holding pre-workshops for service users to learn about methods and discuss experiences so that they are more comfortable with ‘experts’ and can rehearse contributions” - “Be clear about the potential for impact of end-user involvement on the dissemination of findings to enable appropriate management of expectations of all parties involved” - “End-user involvement may be particularly helpful in identifying gaps in the research and developing recommendations for future research. Adequate time to consider not only the potential transferability of findings but also the gaps in the research can extremely be valuable”. | - “Informal training and support were provided by the research team in response to questions from end-users as they arose. E.g., explanation of research methods and terminology were provided both in written documents and during Event 1 and questioning and debate were encouraged”. - “In terms of support, the PenCRU Family Faculty coordinator attended Event 1 and was available to support and update individuals throughout the project” - “The researchers produced a worksheet to aid and focus discussion during Event 2” | 21 months between application for funding and writing of first version of draft |
| 3 | Hyde et al., 2017 | 1. “Researchers and RUG support team planned and organized workshops at three key points in the review process: designing protocol, interpreting results, and planning dissemination of findings” 2. “RUG members were offered reimbursement for their time and travel” | 1. “To inform the systematic review” 2. “To ensure sustainable patient and public involvement in research” | - “Use an established PPIE network if this is possible. It may be easier to recruit people with a different condition using an existing support network” - “Start PPIE as early as possible – that is at question formation stage” - “Recognize that PPIE may extend the research timeline” - “Apply for funding for PPIE and engagement activities, for example members attending conferences” - “Seek advice for funding application” - “Offer reimbursement for time and travel at recommended rates” - “Resources time for PPIE and administrative support” - “Allow for researcher time for developing materials, writing up notes, discussing impact of PPIE” - “Have both a lead researcher and if possible PPIE coordinator” - “Be flexible in how and when members are involved in on-going projects. Make expectations and flexibility clear at the beginning” - “Decide on a minimum of members needed, recruit more than this and allow time for possible rearrangement of meetings” - “Encourage continued participation, agree realistic outcomes initially and give updates on progress” - “Consider members who have already successfully worked together” - “Recognize power relations can be an issue to manage. Clearly recognize and appreciate PPIE members expertise” - “Consider researcher training in small group techniques and debrief with any facilitators after meetings” - “Allow time before, during and after meetings for members and researchers to discuss on asocial level and raise any concerns” - “Give members different ways of expressing their opinion and any concerns (written, online, within-group, individually)” - “Cite national and expert guidance that PPIE in systematic reviews does not require ethics approval” - “Consider a model of PPIE with specific support, or recruiting members in different ways so members feel comfortable in their role” - “Consider the research and condition experience needed for different aspects of the project, dissemination, and engagement” - “Discuss members’ training needs, recognizing individuals will have different experiences. Consider sharing existing training resources” | - “RUG support team provided written training material”. - “A RUG coordinator and a user support worker supported RUG members and liaised with the research team” - “One researcher worked with the RUG members and support team throughout the systematic review process” | - Three workshops, lasting 3 hours each - PPIE lasted 10 months and beyond |
| 4 | Jamal et al., 2015 | 1. Recruiting young people from existing PPI group 2. Utilizing consensus method during consultative meetings | 1. “To allow [easy] access to an established group of young people already familiar with health research who met frequently enough to seek their views at the decision stages of the review” 2. “To address the inequality of participation and the tendency of open discussion to be dominated by a subset of voices” | N/A | - No training was organized for PPI members (due to non-collaboration with researchers in the review process) | - First consultative meeting lasting just over an hour; held in September 2010 - Second consultation in May 2011 |
| 5 | Oliver et al., 2015 | 1. Recruiting experienced young people from a recognized PEAR group 2. Using a glossary to explain technical terms at the beginning of workshop sessions. 3. Researchers providing an atmosphere where young people felt their views were accepted and valued | 1. “To elicit young people’s view about the ongoing systematic reviews on childhood obesity” 2. “To use terminology of an appropriate level and language, which would have been familiar to [young people’s] age group” 3. “To foster open and easy discussion” | N/A | - “A researcher from the review team trained both groups on systematic reviewing during two meetings” - “Scripts and materials were developed by the research team, in collaboration with the NCB senior researcher leading the PEAR project” - “A glossary was provided, and technical terms were explained at the beginning of the sessions”. | - Two workshops, lasting approximately two and a half hours each |
| 6 | Troya et al., 2019 | 1. “Travel reimbursements and vouchers for compensation of time spent [by PPIE member] were provided after each workshop” 2. “Discussions with PPIE group to clarify roles and refine levels of participation” 3. The research team documented the changes made and overall contributions after each of the workshops and fed this back to PPIE members | 1. “To follow current guidelines [for involving PPIE members]” 2. “To avoid overburdening” 3. “To record the impact of the involvement and contribution of PPIE members in the study” | - “Consideration of PPIE involvement from early stages of planning research (ideally when preparing funding application)” - “Allocate enough time for PPIE involvement and possible delays - realistic deadlines” - “Liaise with PPIE network and have a lead PPIE coordinator” - “Early consideration of PPIE involvement in order to plan and allocate enough time and funding” - “Use available resources for guidance and templates of PPIE and lay friendly language” - “Offer reimbursement to PPIE members” - “Keep clear and accessible records of PPIE involvement throughout the different stages of the research project” - “Clarify involvement and level of involvement by each of the members” - “Value the involvement, contribution, added perspective given by PPIE” - “Liaise with research team and PPIE coordinator to ensure meaningful involvement” - “Provide clear expectations, define roles and responsibilities, involvement timelines” - “Ensure PPIE’s needs are considered, and involvement is not resulting in burdening members” - “Ensure feedback is provided regarding PPIE’s impact and contribution to the study, as well as stage of study” - “Acknowledge the different needs PPIE members may have which can limit their ongoing involvement” - “Liaise with PPIE network to ensure clear, open, accessible, and bilateral communication” - “Provide different communication avenues (post, email, phone)” - “Presenting materials sensitively and cautiously” - “Offer avenues of support in needed (e.g., GPs)” - “Ongoing consideration of physical and emotional needs” - “Ensure members involvement can stop at any point they wish” - “Acknowledgement and consideration of different group dynamics and power balance amongst PPIE members” - “Awareness of ethical issues around safety and well-being” - “Liaise with PPIE network regarding expense guidance” - “Have a clear conversation regarding level of involvement and capacity of PPIE members as well as confidentiality and anonymity possible needs when acknowledging involvement” - “Be prepared for inclusive involvement of members in engagement activities” | - “Support (logistical, training and well-being) was provided by the PPIE coordinator, in addition to the attending research members” - “Logistical support included ensuring meeting venues were accessible to members, as well as coordinating meetings at a date and time convenient for PIE members” - “Training support entailed lay friendly and accessible materials explaining the details of the research project” - “Support for well-being included enabling members to feel they could speak freely within workshops and ask questions of any kind and careful observation of members’ emotional and physical needs (i.e., presenting data sensitively, ensuring adequate breaks for refreshment)” - “Regarding researchers’ well-being, research members had the opportunity to discuss sensitive and potentially upsetting matters with the rest of the research team after each workshop and throughout the study duration”. - “Workshop held with PPIE to discuss the concept and process for undertaking SRs, and time for questions” | - Duration of the study was 3 years - Workshops lasted from 2 to 3 hours (half of this time was allocated to the systematic review component [i.e., one to one and half hours]) |
| 7 | Vale et al., 2012 | 1. “Establishment of a small Reference group” 2. “Evaluation of the experience from both the PRPs and researchers’ perspectives” | 1. “To provide advice on the recruitment of women, provision of support and information, and on the activities they might undertake” 2. “To learn from the experiences of the PRPs and researchers to inform future practice” | - “Set out realistic timelines at the outset of the project” - “Inform PRPs about any delays to progress of the project” - “Provide better information about how the results of meta-analyses are presented” - “Explain to the PRPs about the format of the Collaborators’ meeting, giving them opportunity to opt in or out” - “Researchers planning to involve patients in their research should request additional resources in funding applications” - “Novel researchers should make use of a Reference Group to facilitate patient involvement and to advise researchers” - “Reviewers should try to engage with a relevant patient group or organization when embarking on a new review” | - “Establishment of a small Reference group to develop terms of reference and a role description for PRPs who were to get involved” - “Reference Group provided feedback and comments on a detailed information folder and accompanying workshop aimed at describing systematic reviews and meta-analyses to PRPs” | - No clear project timescale was provided - First meeting with PRP in October 2005 - First Collaborators’ meeting with PRPs held in May 2006 - Second Collaborators’ meeting with PRPs held in July 2006 - PRPs involved in main publication of review from 2007 to 2008 - Final evaluation of PRP involvement in August 2010 |
| 8 | Walker et al., 2021 | 1. “All travel expenses were reimbursed in cash on the day of the meeting, and CYP were given high-street vouchers for each meeting attended” | 1. “To recognize CYP’s time and expertise” | - “Consider the involvement of a dedicated PPIE facilitator” - “Provide clear and accessible information on the purpose of the evidence synthesis, how the synthesis will be carried out and how PPIE will be incorporated” - “Foster a shared motivation to co-produce meaningful, accessible, and useful findings”. - “Maintain flexibility in approach to accommodate changing needs and demands on the PPIE group”. - “Enable flexibility in methods of contribution, for example email, telephone and face to face” - “Maintain contact between meetings”. - “Regularly check-in with individuals to ensure that the involvement is mutually beneficial for them and the research” - “Maintain realistic expectations regarding the potential impact of PPIE contributions on the project” - “Provide opportunities for members of the PPIE group to take ownership of the work by taking part in the dissemination of findings, for example by attending conferences, making podcasts, and writing blog posts and plain language summaries” | No clear information on CYP training | - A 15-month project (January 2016 to April 2017) – four meetings held on 2^nd^, 9^th^, 13^th,^ and 15^th^ months of the project |

Abbreviation: PRP, patient research partner; EuroTEAM, Towards Early biomarkers in Arthritis Management; PARE, People with Arthritis/Rheumatism in Europe; RA, rheumatoid arthritis; PPI, patient and public involvement; ADHD, attention deficit hyperactivity disorder; RUG, research user group; PPIE, patient and public involvement and engagement; ALPHA, Advice Leading to Public Health Advancement; NCB, National Children’s Bureau; PEAR, ‘Public health, Education, Awareness, Research’; CYP, children and young people; LTC, long-term condition; CYPAG, Children and Young People’s Advisory Group
