# Supplementary material for: The benefits, challenges, and best practice for patient and public involvement in evidence synthesis: A systematic review and thematic synthesis
Source: Health Expect. 2023 Jun 1;26(4):1436–52. doi: 10.1111/hex.13787 (PMC10349234; doi:10.1111/hex.13787)
Supplement: Supplementary file 6 — Supporting information. [file HEX-26--s006.docx]

**Supplementary File 6: Summary of themes/concepts on the benefits, challenges, and best practice for PPI in** **evidence synthesis/systematic review projects**

**Supplementary File 6a. Benefits described by patients/public**

| **Themes/concepts** | **Examples of codes** | **Studies contributing to the theme/concept** |
| --- | --- | --- |
| Gaining knowledge | Acquiring new skills  Acquiring knowledge  Improving personal skills  Gaining experience  Sharing a learning experience  Discovering pieces of information  Sharing ideas  Sharing useful information  Identifying useful information  Adding an unknown viewpoint | Bayliss et al., 2016; Vale et al., 2012; Walker et al., 2021 |
| Empowerment | Improving confidence  Shaping future attitude through experience  Open exploration of beliefs and perceptions  Feeling a sense of achievement  Exceeding personal expectations  Highlighting others’ concerns  Making positive changes  Feeling empowered  Creating empowering relationships  Hearing others’ perspectives  Gaining assistance from others  Making valued contributions | Bayliss et al., 2016; Coon et al., 2016; Vale et al., 2012; Walker et al., 2021 |

**Supplementary File 6b. Challenges described by patients/public**

| **Themes/concepts** | **Examples of codes** | **Studies contributing to the theme/concept** |
| --- | --- | --- |
| Poor communication | Unclear use of language  Inadequate feedback and support  Difficulty in understanding information  Being unsure about assigned tasks  Inadequate training  Lack of preparation | Bayliss et al., 2016; Coon et al., 2016; Vale et al., 2012 |
| Time | Not having enough time  Having a short deadline  Meeting unrealistic deadline  Late evaluation of project  Lack of preparation  Proposing early evaluation of project  Criticizing longer project duration  Losing personal interests over time  Insufficient preparation | Bayliss et al., 2016; Vale et al., 2012 |
| Low self-esteem | Self-doubt regarding involvement  Not giving credit to self  Being critical about self  Recalling painful experience  Experiencing negative emotions | Bayliss et al., 2016; Coon et al., 2016; Vale et al., 2012 |

**Supplementary File 6c. Benefits described by researchers**

| **Themes/concepts** | **Examples of codes** | **Studies contributing to the theme/concept** |
| --- | --- | --- |
| Improving quality | Identifying gaps in evidence  Informing the search strategy  Identifying relevant outcomes  Establishing potential transferability of findings  Critiquing themes  Informing data analysis  Opportunity to share findings  Revisiting data synthesis  Defining the review question  Defining aims and design for involvement  Supporting the review and involvement  Amending review protocol  Amending data extraction forms  Developing framework for synthesis  Shaping the research process  Informing decisions  Interpreting the literature  Ensuring continuity in research process | Coon et al., 2016; Hyde et al., 2017; Jamal et al., 2015; Oliver et al., 2015; Troya et al., 2019; Vale et al., 2012; Walker et al., 2021 |
| Enhancing relevance | Confirming relevance of findings  Exploring knowledge and experience  Identifying relevant outcomes  Raising ideas about relevance of literature  Incorporating patient priorities  Identifying patient perspective and priorities  Prioritizing members’ views  Improving confidence  Improving understanding  Adding an unknown viewpoint  Prioritizing public needs  Addressing real-world concerns  Reflecting personal experience | Coon et al., 2016; Hyde et al., 2017; Jamal et al., 2015; Oliver et al., 2015; Troya et al., 2019; Vale et al., 2012; Walker et al., 2021 |
| Enhancing dissemination of findings | Targeting results at practitioners  Participating in dissemination of findings  Publishing editorial with patients  Drafting the editorial | Hyde et al., 2017; Troya et al., 2019; Vale et al., 2012; Walker et al., 2021 |

**Supplementary File 6d. Challenges described by researchers**

| **Themes/concepts** | **Examples of codes** | **Studies contributing to the theme/concept** |
| --- | --- | --- |
| Time | Having a tight deadline  Late involvement of members  Having little time  Time pressures  Lack of time  Inadequate time  Time demands | Coon et al., 2016; Hyde et al., 2017; Oliver et al., 2015; Troya et al., 2019; Vale et al., 2012 |
| Balancing inputs and managing relations | Managing expectations  Balancing enthusiasm  Being uncomfortable  Managing emotions  Conflict surrounding outcomes  Balancing relationships  Addressing mutual benefits  Highlighting relationship issues  Struggling to build good relationships  Limited opportunities for collaboration  Managing group dynamics  Inadequate opportunity for input  Feeling disappointed  Feeling worried  Feeling hopeful  Determining needs and priorities  Establishing need for involvement  Accommodating health needs  Discussing sensitive topics | Coon et al., 2016; Hyde et al., 2017; Troya et al., 2019; Vale et al., 2012; Walker et al., 2021 |
| Resources and training | Funding  Having inadequate skills  Managing gaps in communication  Poor recruitment strategy  Inappropriate communication medium  Not utilizing engagement medium  Unrecognizable contribution  Lack of congruence  Avoiding tokenistic involvement  Clear and open communication  Having a defined budget | Coon et al., 2016; Hyde et al., 2017; Jamal et al., 2015; Troya et al., 2019; Vale et al., 2012; Walker et al., 2021 |

**Supplementary File 6e. Authors’ recommendations for best practice**

| **Themes/concepts** | **Examples of codes** | **Studies contributing to the theme/concept** |
| --- | --- | --- |
| Provision of sufficient time and resources | Providing adequate time  Asking about time commitment  Considering timings for involvement  Planning for longer project duration  Setting realistic deadlines  Early consideration of PPI involvement  Providing project timelines  Providing adequate resources  Assessing costs and benefits of PPI  Applying for funding  Seeking advice on funding  Offering reimbursement to PPI  Requesting additional resources | Bayliss et al., 2016; Coon et al., 2016; Hyde et al., 2017; Troya et al., 2019; Vale et al., 2012 |
| Developing a clear recruitment plan | Planning involvement  Planning recruitment strategies  Clarifying level of involvement  Providing recruitment options  Using an established PPI network  Having a lead PPIE coordinator  Using a Reference group  Engaging with relevant group  Using an established PPI network | Coon et al., 2016; Hyde et al., 2017; Troya et al., 2019; Vale et al., 2012; Walker et al., 2021 |
| Provision of sufficient training and support | Providing adequate training  Providing adequate support  Identifying training needs  Identifying support needs  Evaluating training sessions  Clarifying PPI roles  Obtaining feedback about training materials  Sharing training resources  Developing training materials  Providing feedback to PPI  Need for proper medium of communication  Using focus groups with appropriate numbers  Providing different communication avenues  Explaining format for meetings | Bayliss et al., 2016; Coon et al., 2016; Hyde et al., 2017; Troya et al., 2019; Vale et al., 2012; Walker et al., 2021 |
| Need to foster positive working relationships | Identifying value of PRP inputs  Monitoring and evaluation of PRP inputs  Clarifying impacts of involvement  Resolving conflicts between PPI members  Recognizing power relations  Recognizing and appreciating PPI  Being sensitive and cautious  Maintaining relationships with PPI  Creating shared motivations | Bayliss et al., 2016; Coon et al., 2016; Hyde et al., 2017; Troya et al., 2019; Walker et al., 2021 |
